# Feedback-regulated 3D-printed hydrogel micropatch for integrated therapy of fungal keratitis

**DOI:** 10.7150/thno.134232

**Published:** 2026-08-24

**Authors:** Yujie Liu, Li Ma, Guodong Fang, Tan Li, Hongran Zhao, Ting Wu, Jiajia Zhang, Xia Qi, Xiangyue Hu, Hongwei Wang, Chao Yang, Depeng Shi

**Affiliations:** 1 Eye Institute of Shandong First Medical University, State Key Laboratory Cultivation Base, Shandong Provincial Key Laboratory of Ophthalmology, Qingdao Eye Hospital of Shandong First Medical University, Qingdao 266071, China.; 2 Department of Ophthalmology, First Affiliated Hospital of Dalian Medical University, Dalian 116011, China.; 3 College of Materials Science and Engineering, Qingdao University, Qingdao, Shandong, 266071, China.

**Keywords:** fungal keratitis, dual-network hydrogel, multifunctional corneal micropatch, cerium metal-organic frameworks, 3D printing

## Abstract

**Rationale:**

Fungal keratitis remains clinically challenging due to limited drug bioavailability, frequent dosing, reactive oxygen species (ROS)-mediated stromal damage, perforation risk, and bacterial coinfection. In the study, we developed a feedback-regulated multifunctional corneal micropatch (MFCP) via 3D printing for the treatment of fungal keratitis.

**Methods:**

The dual-network hydrogel micropatch comprises an ROS-responsive hydrogel (RRH), voriconazole-loaded F127DA micelles (VCZ-F127DA), and gatifloxacin-loaded cerium metal-organic frameworks (GAT-MOFs). We characterized its physicochemical properties and evaluated its biocompatibility, corneal healing capacity, ROS scavenging activity, and antimicrobial performance. Histological staining, cytokine assays, and transcriptomic sequencing were used to assess its therapeutic efficacy in a mouse model of fungal keratitis.

**Results:**

MFCP exhibits tunable curvature, high transparency, and mechanical properties matching those of the cornea, along with good biocompatibility. High ROS level can accelerate breakdown of the micropatch and promote drug release, generating strong synergistic antifungal and antibacterial activities. The released GAT-MOFs scavenge excessive ROS, forming a negative feedback loop. In the mouse model of fungal keratitis, MFCP mitigates corneal infection, lowers proinflammatory cytokines, and accelerates tissue repair, with therapeutic efficacy superior to that of voriconazole eye drops. Transcriptomic analysis reveals suppression of NF-κB-mediated inflammatory signaling and upregulation of corneal regenerative pathways after treatment with MFCP.

**Conclusion:**

These findings demonstrate that MFCP represents a viable therapeutic option for fungal keratitis.

## Introduction

Fungal keratitis, mainly caused by *Fusarium solani* (*F. solani*) and *Aspergillus fumigatus*, is a sight-threatening corneal infectious disease 1, 2. It is a leading cause of corneal blindness worldwide, with a much higher burden in developing regions 3, 4. Multiple clinical and behavioral factors have driven its steadily climbing incidence. Several factors have been linked to fungal keratitis and its rising global incidence, including ocular trauma, contact lens wear, ophthalmic surgery, and prolonged glucocorticoid use 5-9.

The early treatment of fungal keratitis mainly relies on topical antifungal eye drops 10-12. Treatment outcomes are often compromised by rapid precorneal clearance and limited bioavailability 13-15. Advanced carriers, such as hydrogels and nanomedicines, improve ocular drug retention and bioavailability 16-18. However, most existing platforms offer only passive sustained release and cannot adapt to progressive pathological changes 19. This passive release strategy increases the risk of antifungal resistance. A feedback-regulated delivery platform that adjusts drug release to disease dynamics could overcome these limitations 20.

Reactive oxygen species (ROS) generated by host immune activation can damage the corneal stroma, potentially causing thinning, perforation, and vision loss 21-24. Bacterial coinfection worsens the prognosis of fungal keratitis, with enucleation rates up to 21% in some reports 25-27. Antifungal monotherapy alone is insufficient 16, 28, 29. Even after infection and inflammation subside, residual stromal defects can still perforate. 3D-printed hydrogel patches offer a way to fill ulcerated areas, stabilize the cornea, and support healing 30, 31. A 3D-printed patch combining ROS scavenging, antibacterial capacity, healing promotion, and stromal reinforcement could meet multiple needs in fungal keratitis management 32.

**Scheme 1:**
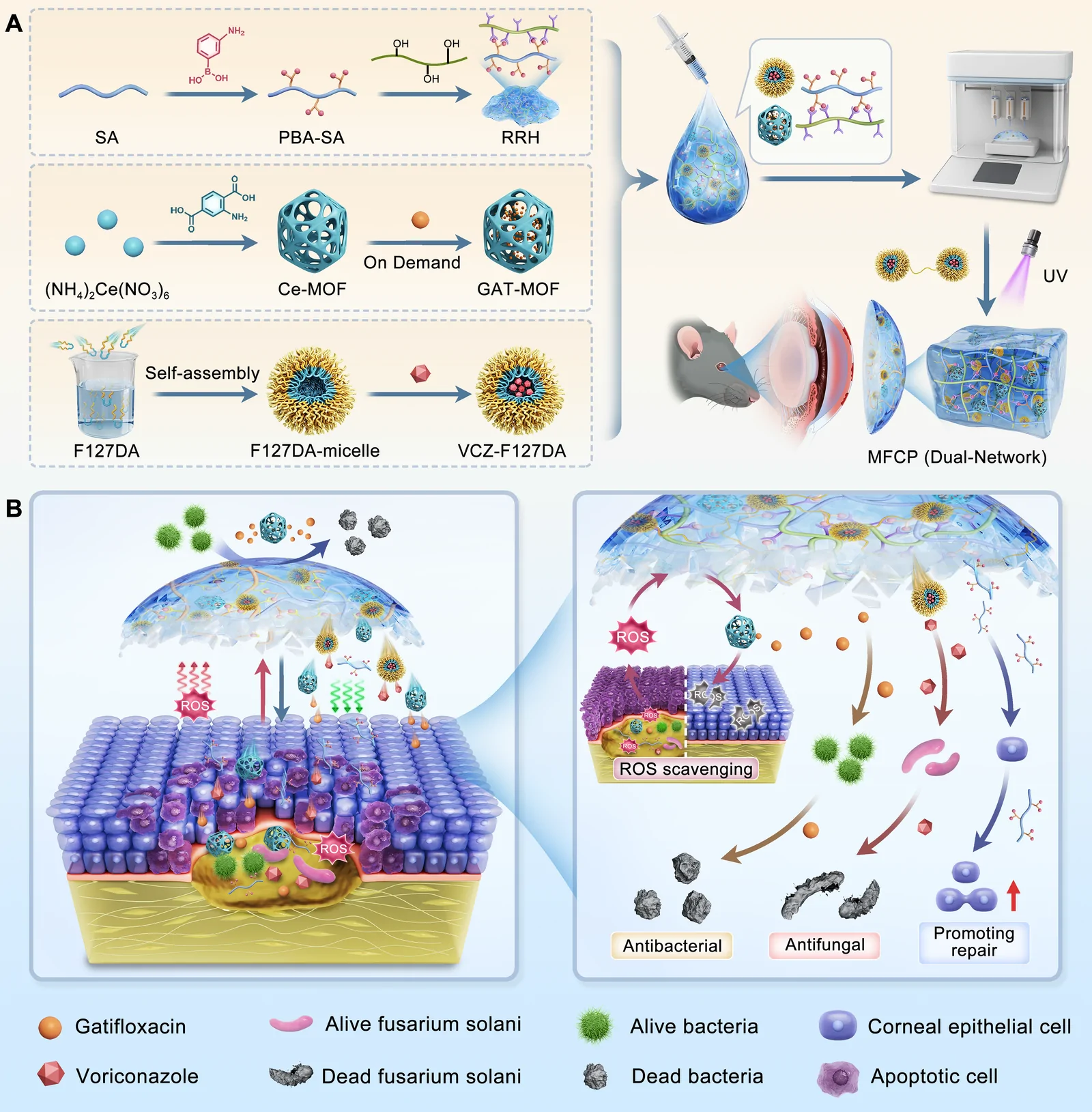
Fabrication and mechanisms of multifunctional corneal micropatch (MFCP) for fungal keratitis. (A) Fabrication of MFCP from ROS-responsive hydrogel (RRH), voriconazole-loaded F127DA micelles (VCZ-F127DA), and gatifloxacin-loaded metal-organic frameworks (GAT-MOFs) by 3D printing. (B) Proposed mechanisms of action of the MFCP in fungal keratitis: ROS-responsive degradation, ROS scavenging by MFCP (forming a negative feedback loop), and antibacterial, antifungal, and promoting repair.

In this study, a multifunctional corneal micropatch (MFCP) was developed for feedback-regulated treatment of fungal keratitis (Scheme 1). The patch was fabricated via 3D printing using a bioink, which was composed of an ROS-responsive hydrogel (RRH), voriconazole-loaded F127DA micelles (VCZ-F127DA), and gatifloxacin-loaded cerium metal-organic frameworks (GAT-MOFs, prepared by facile stirring and enabling sustained and tunable release). High ROS levels cleave boronate ester bonds within the patch, triggering degradation and a burst of drug release to fight acute infection. The released GAT-MOFs scavenge ROS and curb inflammation; as ROS decline, degradation slows and release tapers, forming a self-regulating cycle. In addition, ROS-responsive release, antimicrobial activity, antioxidant effects, and healing promotion of MFCP were evaluated, and transcriptomics was used to investigate its mechanism against fungal keratitis.

## Methods

### Preparation of GAT-MOFs

Cerium metal-organic frameworks (Ce-MOFs) were prepared by reacting a solution of (NH_4_)_2_Ce(NO_3_)_6_ (822 mg in 3 mL deionized water) with acetic acid (3 mL), anhydrous ethanol (20 mL), and 2-aminoterephthalic acid (272 mg) 33. The reaction mixture was maintained under continuous stirring at 25 °C for 2 h. The solid was pelleted by centrifugation, rinsed with ethanol and H₂O, followed by drying in vacuo at 60 °C overnight. These synthesized Ce-MOFs (10 mg) were then used for drug loading by codispersing with GAT (30 mg) in deionized water (10 mL) and stirring at 4 °C for 24 h. After centrifugation and removal of the unloaded drug in the supernatant, the solid was dried under vacuum at low temperature to yield the GAT-MOFs.

### Fabrication of MFCP

Sodium alginate (SA, 1.0 g) was dissolved in deionized water (100 mL), followed by sequential addition of EDC (0.96 g) and 3-aminophenylboronic acid (0.39 g). The mixture was kept under agitation at 25 °C for 24 h. It was dialyzed against deionized water (MWCO: 3500 Da) over 3 days, followed by lyophilization to yield phenylboronic acid-grafted alginate (PBA-SA). An RRH was formed by mixing 4% (w/v) PBA-SA solution with 4% (w/v) polyvinyl alcohol solution. VCZ-F127DA were prepared by dissolving F127DA at 15% (w/v) in a 0.25% (w/v) lithium phenyl-2,4,6-trimethylbenzoylphosphinate solution at 4 °C 34, adding 1% VCZ solution (w/v), sonicating at 300 W for 5 min under cooling, and incubating at 37 °C for 2 h to facilitate micellization. The bioink was formulated by adding GAT-MOFs (125 µg/mL) to a 1:1 mixture of the RRH and VCZ-F127DA. This bioink was loaded into a 3D bioprinter syringe and extruded according to a G-code file designed based on corneal curvature. The printed structure was subsequently crosslinked under 405 nm UV light for 30 s to yield the MFCP. A dual-network corneal patch (DNCP) was fabricated identically but using the bioink without GAT-MOFs.

### Physicochemical characterization

#### Determination of GAT loading capacity

The content of GAT was quantified via a UV-near-visible light spectrophotometer (UV-3600Plus, Shimadzu, Japan), with a detection wavelength of 405 nm. The unbound GAT in the supernatant was calculated based on the established linear calibration curve A = 0.069C + 0.0416 (R^2^ = 0.9997). The drug loading capacity (DL%) was determined using the formula: DL% = (*W_T_* - *W_S_*) / *W_np_* × 100%, where *W_T_*, *W_S_* and *W_np_* denote total drug, the drug in the supernatant, and the dry particle weight, respectively.

#### Drug release curve of MFCP

Swollen MFCPs were immersed in 1 mL of PBS or H_2_O_2_ solutions (50, 100, or 200 μM) at 37 °C with shaking (60 rpm). At different time points, the release medium was sampled (1 mL) and replaced with the same volume of fresh medium. UV-vis spectrophotometry was used to quantify the cumulative release of GAT and VCZ from each sample.

#### ROS scavenging activity of MFCP

Commercial kits were used to assess ROS scavenging activity of MFCP against H_2_O_2_, hydroxyl radicals (·OH), and superoxide anion (O_2_⁻˙). We suspended MFCP samples in 3 mL of 100 mM H_2_O_2_ or PBS and recorded oxygen levels every 5 min over 30 min using a portable meter to monitor dissolved oxygen.

### Cell experiments

#### *In vitro* biocompatibility study

Human corneal epithelial cells (HCECs) and human corneal fibroblasts (HCFs) were adopted to investigate the biocompatibility of MFCP. Live/dead staining: cells in 6-well plates (1 × 10⁵/well) were incubated with MFCP or its extracts (20–100 mg/mL) for 24 h, then stained. CCK-8: cells in 96-well plates (1 × 10⁴/well) were cultured for 24 h, treated with extracts (same concentrations) for 24 h, and assayed per kit instructions. Profibrotic risk was assessed in HCFs after 24 h exposure to extracts, with α-SMA and TGF-β1 mRNA expression determined via qRT-PCR (normalized to GAPDH).

#### Intracellular ROS detection

Cells were treated for 2 h with PBS, H_2_O_2_ (100 µM), H_2_O_2_ (100 µM) + Ce-MOFs (12.5 µg/mL), or H_2_O_2_ (100 µM) + MFCP extract (100 mg/mL). They were then stained with 10 µM DCFH-DA (10 min, 37 °C), washed, counterstained with Hoechst 33342, and examined by fluorescence microscopy. The ROS fluorescence intensity was quantified using ImageJ software.

#### Cell migration and proliferation experiment

HCECs were seeded in 6-well plates and grown until they reached 90% confluence. We created a linear scratch in each well with a sterile 200-µL pipette tip. After washing off the detached cells, we added fresh medium containing PBS, VCZ (1%), GAT-MOFs (12.5 µg/mL), DNCP extract (100 mg/mL), or MFCP extract (100 mg/mL) to the cultures. At the indicated time points (0, 12, and 24 h), wound images were taken. The wound area was quantified with ImageJ, and the percentage of closure was determined.

### *In vitro* antimicrobial efficacy

#### Antibacterial activity

The antibacterial activity of MFCP against *Staphylococcus epidermidis* (*S. epidermidis*) and *Staphylococcus aureus* (*S. aureus*) was assessed using disk diffusion, colony counting, and live/dead staining 18. In the disk diffusion test, 7-mm-diameter wells were punched in Mueller-Hinton agar and loaded with PBS, GAT, GAT-MOFs, DNCP, or MFCP. The plates were then inoculated with bacteria and incubated for 24 h, after which the inhibition zones were measured. To quantify bacterial survival, suspensions (1 × 10⁷ CFU/mL) were treated with the same agents for 12 h, diluted, plated, and counted after 24 h. Live/dead staining with Calcein-AM/PI allowed visualization of bacterial viability under fluorescence microscopy.

#### Antifungal activity study

*F. solani* (AS 3.1829) was purchased from the China General Microbial Strain Preservation Center (Beijing, China). A spore suspension (1 × 10⁷ CFU/mL) was treated with PBS, 100 µM H_2_O_2_, MFCP (100 mg/mL), or MFCP (100 mg/mL) + 100 µM H_2_O_2_. Disk diffusion, colony counting, and live/dead staining were performed as described above for the antibacterial evaluation.

### Animal experiments

C57BL/6 mice (female, 6-8 weeks old) were purchased from Spafu (Beijing, China). All animal experiments were conducted following ARVO guidelines and received approval from the Ethics Committee of Shandong Eye Institute (SDSYKYJS No. 20250107).

#### Biocompatibility study *in vivo*

The *in vivo* biocompatibility of MFCP was examined in normal mice. All animals were divided into three experimental groups (n = 3). The test group had the MFCP placed on the cornea; the control groups were given topical 1% VCZ drops or PBS, with treatments applied three times daily. On day 7, ocular tolerance was assessed by slit-lamp, optical coherence tomography (OCT), and fluorescein staining, followed by eye enucleation for H&E staining.

#### Corneal wound healing

Corneal wounds were generated in normal mice as described previously 35. After pentobarbital anesthesia, a 2.5 mm epithelial defect was made with an Algerbrush® II rust ring remover. The mice were randomized into five groups (n = 3) and administered with DNCP, MFCP, GAT-MOFs (12.5 µg/mL), 1% VCZ eye drops, or PBS. The DNCP and MFCP were applied once, whereas all other treatments were given topically three times daily. Corneal wound healing was monitored by fluorescein staining and bright-field imaging at 0, 24 and 48 h post-injury.

#### *In vivo* antifungal activity study

Fungal keratitis model was induced with mice using the previously reported procedures 36, 37. Corneal wounds were created as described above. Sterile filter disks (2 mm) soaked with *F. solani* (1 × 10⁷ CFU/mL) were placed on the epithelial defect, and the eyelids were sutured closed for 24 h. Model validity was assessed using a standardized scoring system. Mice with established fungal keratitis were randomly divided into five groups: PBS, 1% VCZ eye drops (three times daily), 1% VCZ + 0.3% GAT eye drops (each instilled three times daily), DNCP and MFCP. Clinical severity scores based on the scoring system were recorded on days 1, 3, 5, and 7 (n = 6).

On day 3, corneas were harvested for fungal burden assessment via calcofluor white staining, with ImageJ measuring mycelial invasion depth (n = 3). On day 7, corneas were collected for H&E staining (n = 3), cytokine quantification (IL-1β, IL-6, and TNF-α) (n = 3), and transcriptomic analysis (n = 3). The antifungal effect of MFCP on corneal tissue was assessed by colony counting. Treated eyes were sampled on days 1, 3, 5, and 7 (n = 3 each). The collected corneal tissues were homogenized, serially diluted, and plated on Sabouraud dextrose agar; fungal colonies were enumerated after incubation to determine the residual fungal burden.

### Statistical analysis

Data are presented as mean ± standard deviation. All experiments were repeated at least three times. Statistical analysis was performed using one-way analysis of variance or Student's t-test (GraphPad Prism 8). P < 0.05 was considered statistically significant (*P < 0.05, **P < 0.01, ***P < 0.001).

## Results and Discussion

### Characterization of GAT-MOFs

**Figure 1:**
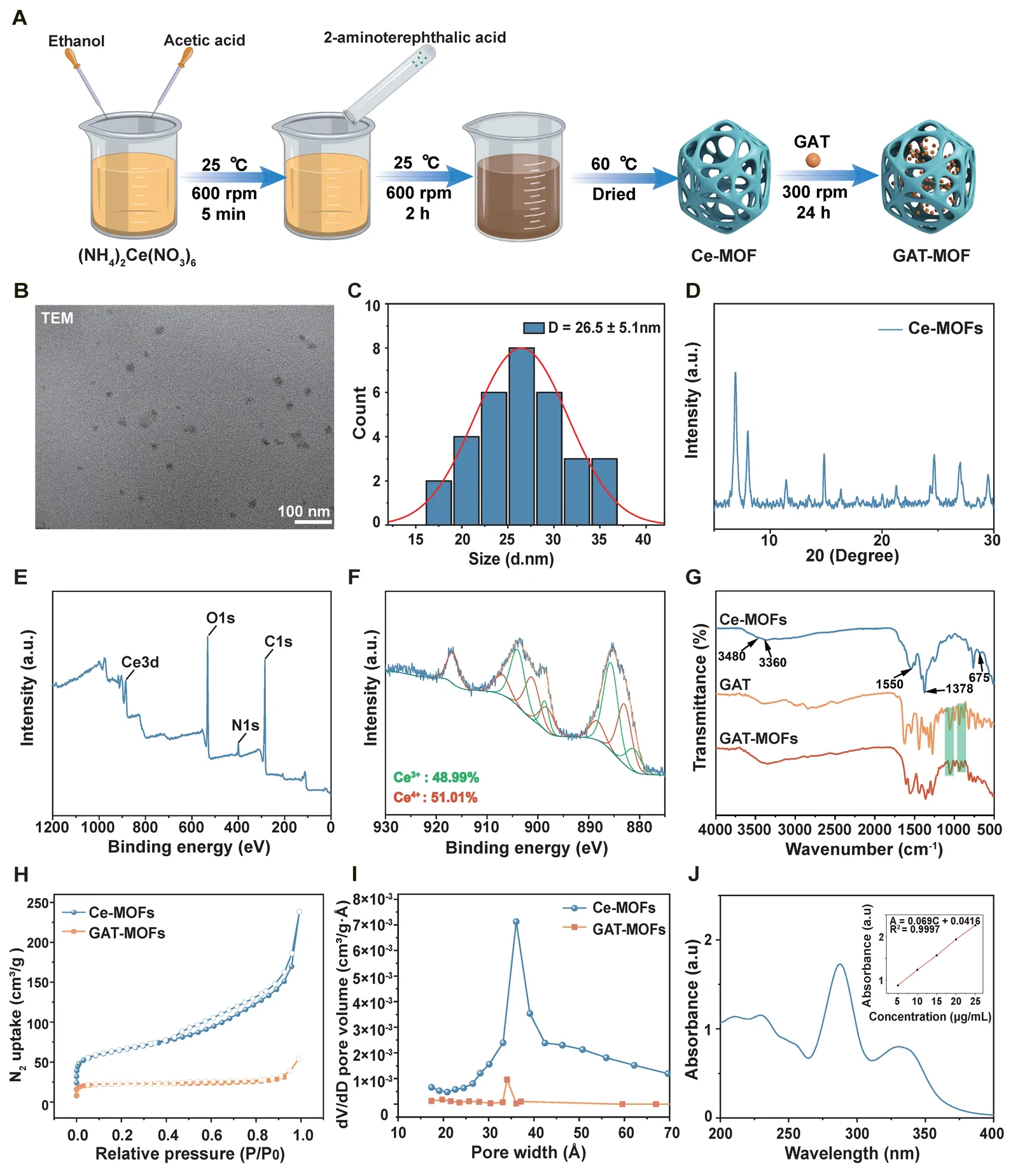
Characterization of GAT-MOFs. (A) Synthesis process of GAT-MOFs. (B) TEM micrograph, (C) particle size analysis, (D) XRD patterns, (E) XPS spectrum, and (F) Ce 3d XPS spectrum of Ce-MOFs. (G) FTIR, (H) BET surface area, (I) pore-size distribution of Ce-MOFs and GAT-MOFs. (J) Drug loading capacity of GAT-MOFs.

Ce-MOFs were prepared at room temperature from ammonium cerium nitrate and 2-aminoterephthalic acid, followed by GAT loading (Figure 1A). SEM showed uniform particles with tens of nanometers in size, and EDS confirmed the presence of C, N, O, and Ce in the nanoparticles (Figure S1). TEM revealed well-dispersed nanoparticles with an average size of 26.5 ± 5.1 nm (Figures 1B-C). XRD exhibited characteristic peaks at 6.9° and 8.0° (Figure 1D), consistent with the previously reported Ce-UiO-66 33. XPS survey spectra revealed Ce, O, C, and N signals, and high-resolution Ce 3d spectra showed both Ce^3+^ and Ce^4+^ in a mixed-valence state (Figures 1E-F). This redox-active couple is expected to confer ROS-scavenging activity, as reported for analogous Ce-based systems 38, 39.

FTIR confirmed the incorporation of amino groups into Ce-MOFs (Figure 1G). The spectrum exhibited Ce-O stretching at 675 cm^-1^ and typical carboxylate bands at 1550 and 1378 cm^-1^. The peaks at 3360 and 3480 cm^-1^ confirmed the N-H stretching of the amino groups. These amino groups may form hydrogen bonds with carboxyl/hydroxyl groups on the ocular surface 40. The positive zeta potential of +1.71 mV (Figure S2) should promote adhesion to the negatively charged cornea via electrostatic attraction 41. These properties should prolong ocular retention and improve drug utilization.

FTIR confirmed GAT loading into Ce-MOFs (Figure 1G). BET showed a specific surface area of 215.48 m^2^/g for pristine Ce-MOFs, with pore sizes predominantly in the 2.3–4.2 nm range (Figure 1H). After GAT loading, the surface area decreased to 69.76 m^2^/g, and the pore-size distribution narrowed markedly (Figure 1I), consistent with pore filling by the drug. UV-vis spectroscopy quantified the loading capacity at 29.6% ± 2.1% (Figure 1J).

### Characterization of MFCP

**Figure 2:**
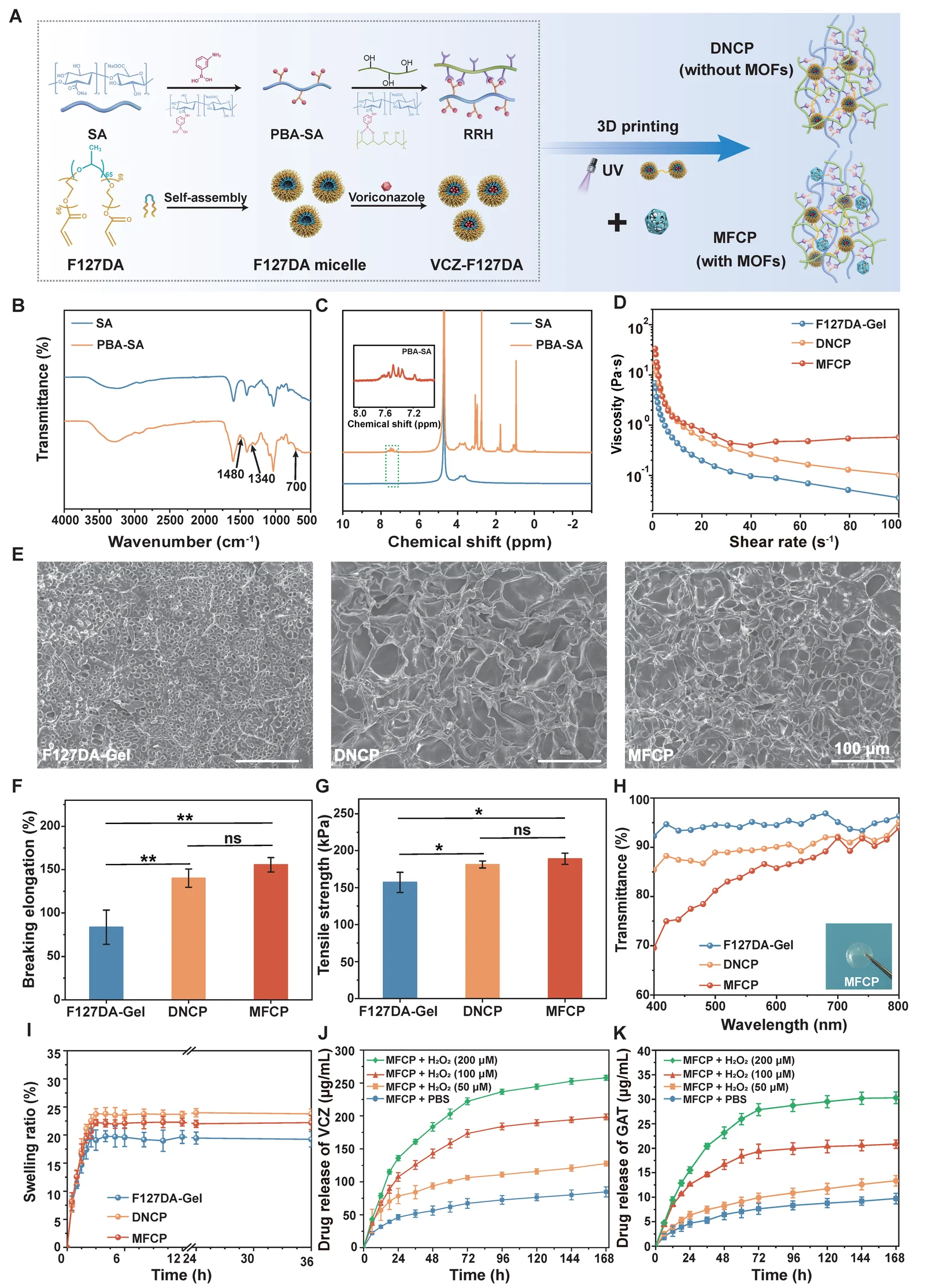
Characterization of MFCP. (A) Synthesis routine of MFCP. (B) FTIR and (C) ^1^H NMR spectra of PBA-SA. (D) Shear-thinning test, (E) SEM, (F) breaking elongation, (G) tensile strength, (H) light transmittance, (I) swelling ratio of light-crosslinked F127DA hydrogel (F127DA-Gel), double-network hydrogel corneal micropatch without GAT-MOFs (DNCP), and MFCP. (J) *In vitro* release curves of voriconazole (VCZ) and (K) gatifloxacin (GAT) from MFCP. *P < 0.05, **P < 0.01, ***P < 0.001.

The MFCP was fabricated using extrusion-based 3D printing with a bioink formulated from RRH, GAT-MOFs, and VCZ-F127DA (Figure 2A). PBA-SA was the key component conferring ROS-responsiveness to the system. Compared with unmodified SA, PBA-SA showed new FTIR peaks at 1480, 1340, and 700 cm^-1^ (Figure 2B). The bands at 1480, 1340, and 700 cm^-1^ are assigned to benzene C=C stretching, boronic acid B-O stretching, and aromatic C-H out-of-plane bending, respectively. The ^1^H NMR spectrum revealed aromatic proton signals at 7.1–7.7 ppm (Figure 2C), confirming conjugation of phenylboronic acid groups.

F127DA, a biocompatible hydrophobic drug carrier, forms a stable hydrogel (F127DA-Gel) via UV-triggered photo-crosslinking in the presence of a photoinitiator 34. Rheological analysis revealed solid-like elastic behavior of all hydrogels, including F127DA-Gel, the DNCP, and the MFCP. As shown in Figure S3, the storage modulus (G′) of these hydrogels consistently exceeded their loss modulus (G″) throughout the tested frequency range. All three hydrogels were shear-thinning, while MFCP showed higher viscosity than F127DA-Gel and DNCP (Figure 2D). SEM revealed the morphology of F127DA-Gel, DNCP and MFCP (Figure 2E).

Beyond conventional casting and molding methods, 3D printing provides structural versatility, and allows precise fabrication of tunable porosity, layer-specific mechanical gradients, and anisotropic architectures 42-44. These features have been associated with therapeutic benefits, including mechanical support, controlled drug release, and cell infiltration. The micropatch can be fabricated by 3D printing to match the corneal curvature for surface fit (Figure S4). Tensile testing revealed that MFCP exhibits an elongation at break of 155 ± 10% and a fracture strength of 189 ± 15 kPa, exceeding those of the F127DA-Gel (Figures 2F-G) 44. These features are essential for temporary support and perforation prevention in infected corneas 45. MFCP exhibited high transparency in the visible range of 400–800 nm, ensuring no visual obstruction after attachment to the ocular surface (Figure 2H). Its equilibrium water content of 88.8 ± 1.4% (Figure S5) and swelling ratio of 22.2 ± 0.5% (Figure 2I) in PBS closely resemble those of the natural corneas. This allows the micropatch to maintain corneal surface moisture and promote epithelial repair 46.

We measured VCZ and GAT release from MFCP under PBS and H_2_O_2_ (50, 100, 200 µM) conditions (Figures 2J-K). The results demonstrate a slow and sustained release profile in PBS. Higher release rates were observed under H_2_O_2_ conditions mimicking severe infection, with a burst observed within 24 h. The release rate decelerated over time, as the H_2_O_2_ concentration was progressively reduced due to the scavenging of Ce-MOFs. The on-demand release behavior can be attributed to the ROS-sensitive cleavage of dynamic borate ester bonds in the PBA-SA network, and is subsequently downmodulated as the scavenging of ROS by Ce-MOFs. These findings suggest that MFCP functions as a ROS-responsive delivery system rather than a passive drug depot.

### Biocompatibility evaluation

**Figure 3:**
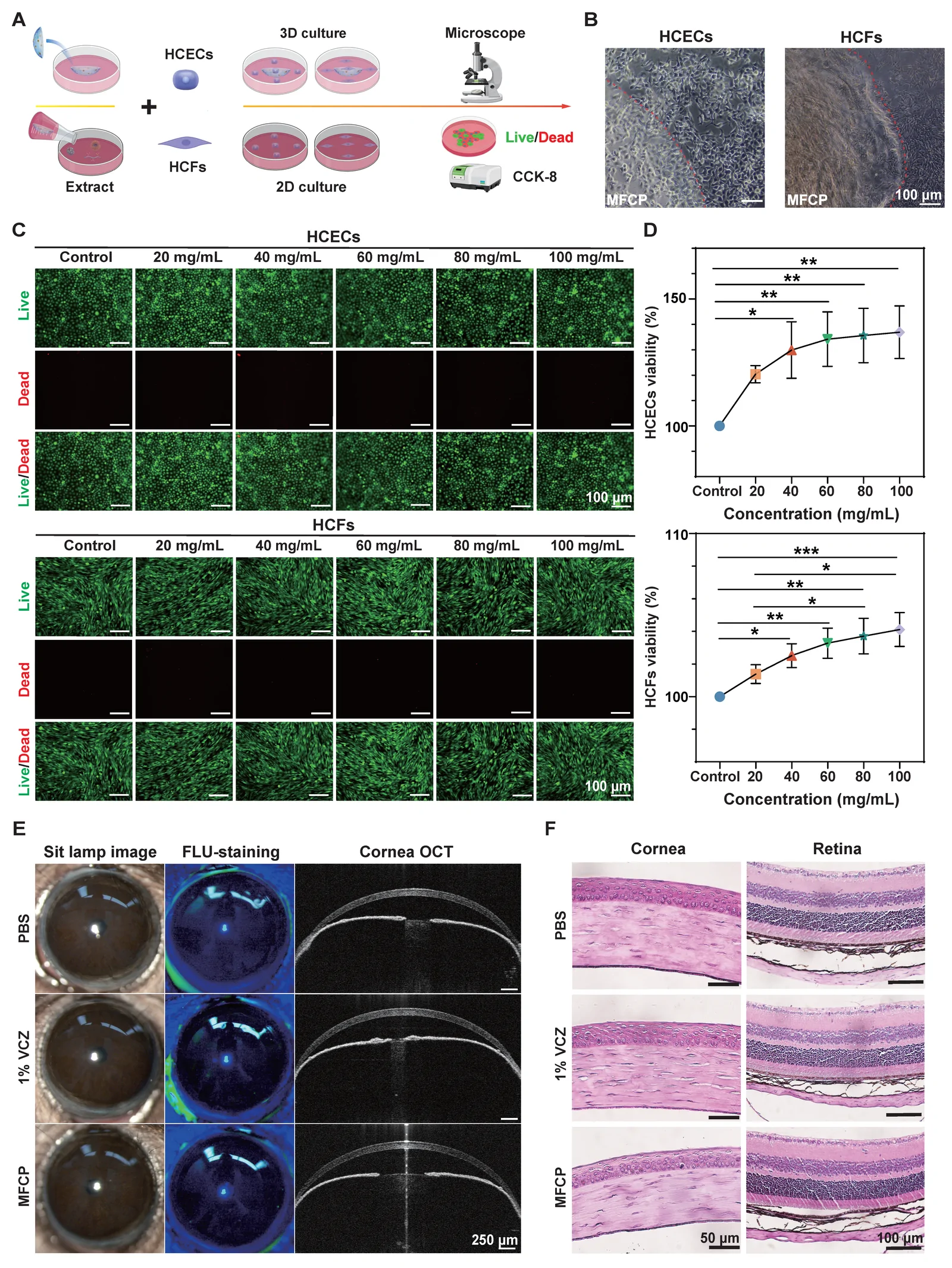
Biocompatibility evaluation of MFCP. (A) Schematic illustration of the cell culture experiment. (B) Coculture model of HCECs and HCFs on MFCP. (C) Viability assessment of corneal cells exposed to MFCP extracts via live/dead and (D) CCK-8 assays. (E) C57BL/6 mouse corneas and (F) H&E-stained corneal/retinal sections following treatment with PBS, 1% VCZ, or MFCP (n = 3). *P < 0.05, **P < 0.01, ***P < 0.001

We evaluated the safety of MFCP through *in vitro* and *in vivo* biocompatibility tests. The cytocompatibility of MFCP was assessed using 3D and 2D cocultures of HCECs and HCFs (Figure 3A). Both cell types adhered to and proliferated on the MFCP surface (Figure 3B). Live/dead staining with MFCP extracts at different concentrations showed no significant increase in cell death for either cell type (Figure 3C). Fluorescence-based live/dead staining (Figure S6) and CCK-8 assays (Figure 3D) confirmed that MFCP extracts did not reduce HCECs or HCFs viability. Instead, a dose-dependent increase in cell proliferation was observed. α-SMA and TGF-β1 mRNA levels were also measured in HCFs treated with MFCP extracts (Figure S7). MFCP did not upregulate either gene at any tested concentration. This indicates it does not drive HCFs toward a myofibroblast phenotype, suggesting a low risk of corneal scar formation.

We placed MFCP to the corneal surface of healthy C57BL/6 mice over a 7-day period, with PBS and 1% VCZ eye drops as controls. There was no conjunctival hyperemia, edema, or corneal opacity in any group by slit-lamp and OCT examinations (Figure 3E), and no obvious inflammatory cell infiltration in the corneal stroma of all groups by H&E staining (Figure 3F). No obvious toxicity or inflammation was observed *in vivo*, suggesting that MFCP is well tolerated and suitable for further evaluation in fungal keratitis.

### Promotion of corneal wound healing

**Figure 4:**
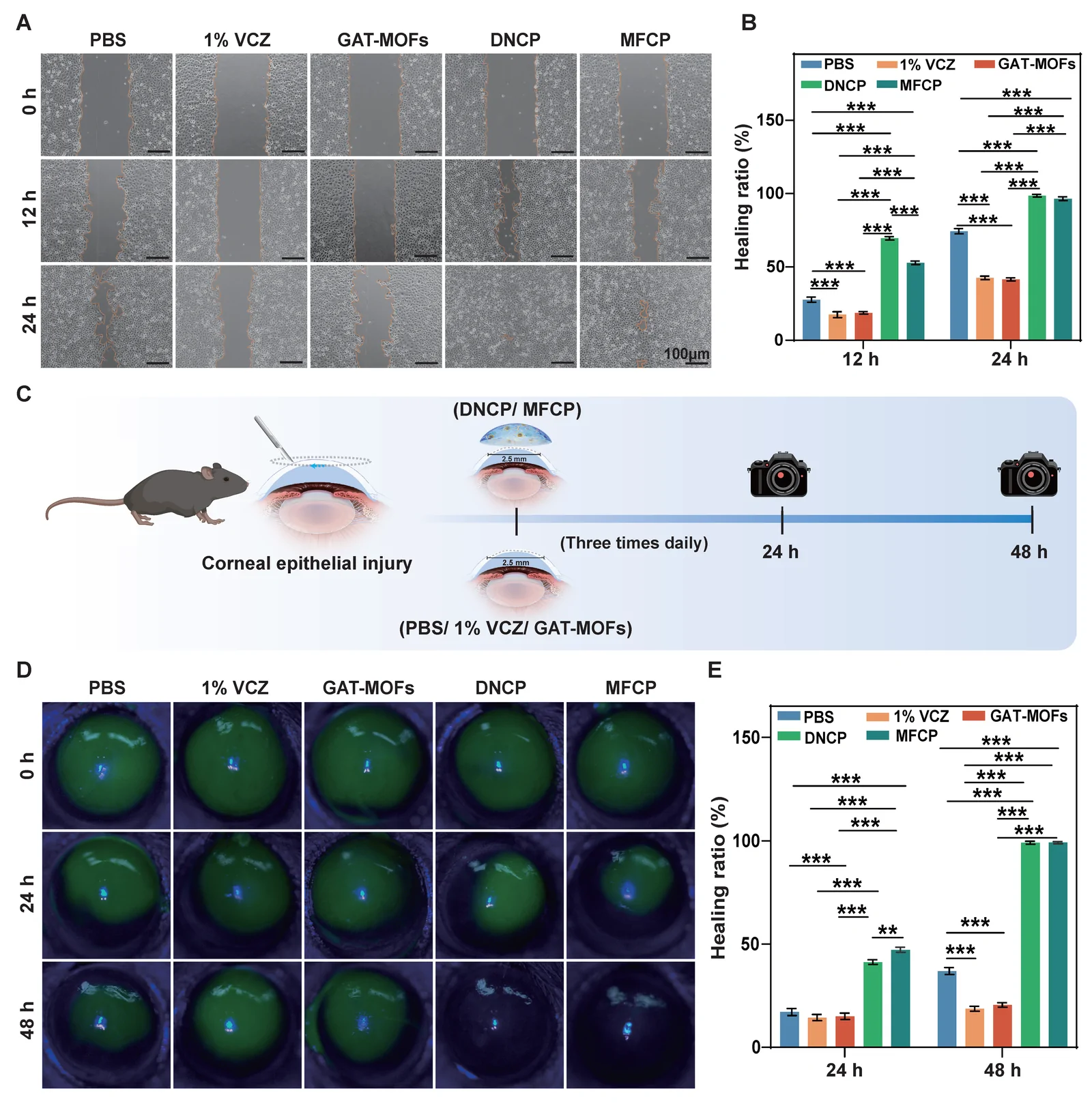
Promotion of corneal wound healing by MFCP. (A) Representative images and (B) quantification of cell scratch assay. (C) Schematic of the mouse corneal wound model. (D) Slit-lamp images and (E) corneal wound healing rates at 0, 24, and 48 h (n = 3). *P < 0.05, **P < 0.01, ***P < 0.001.

We assessed cell migration and proliferation using *in vitro* scratch assays (Figure 4A). Quantitative analysis showed that DNCP and MFCP extracts accelerated scratch closure compared with the other groups at 12 and 24 h (Figure 4B). DNCP had a slightly higher closure rate than MFCP at 12 h, possibly due to the cytotoxic effect of GAT released from MFCP 47. By 24 h, the closure rates of DNCP and MFCP were similar. The superior scratch closure in the DNCP and MFCP groups likely arises from alginate resulting from degradation of the micropatch, which promotes cell migration and proliferation 48.

Corneal wound healing was assessed *in vivo*, and wound closure was tracked by slit-lamp imaging at 0, 24, and 48 h (Figure 4C). Nearly complete re-epithelialization occurred within 48 h in the DNCP and MFCP groups, faster than in the PBS group (Figures 4D and S8). MFCP promoted faster closure than DNCP at 24 h (Figure 4E). This enhanced *in vivo* performance likely reflects the antioxidant capacity of Ce-MOFs, which scavenge ROS and alleviate oxidative stress, accelerating healing 49.

### ROS scavenging activity

**Figure 5:**
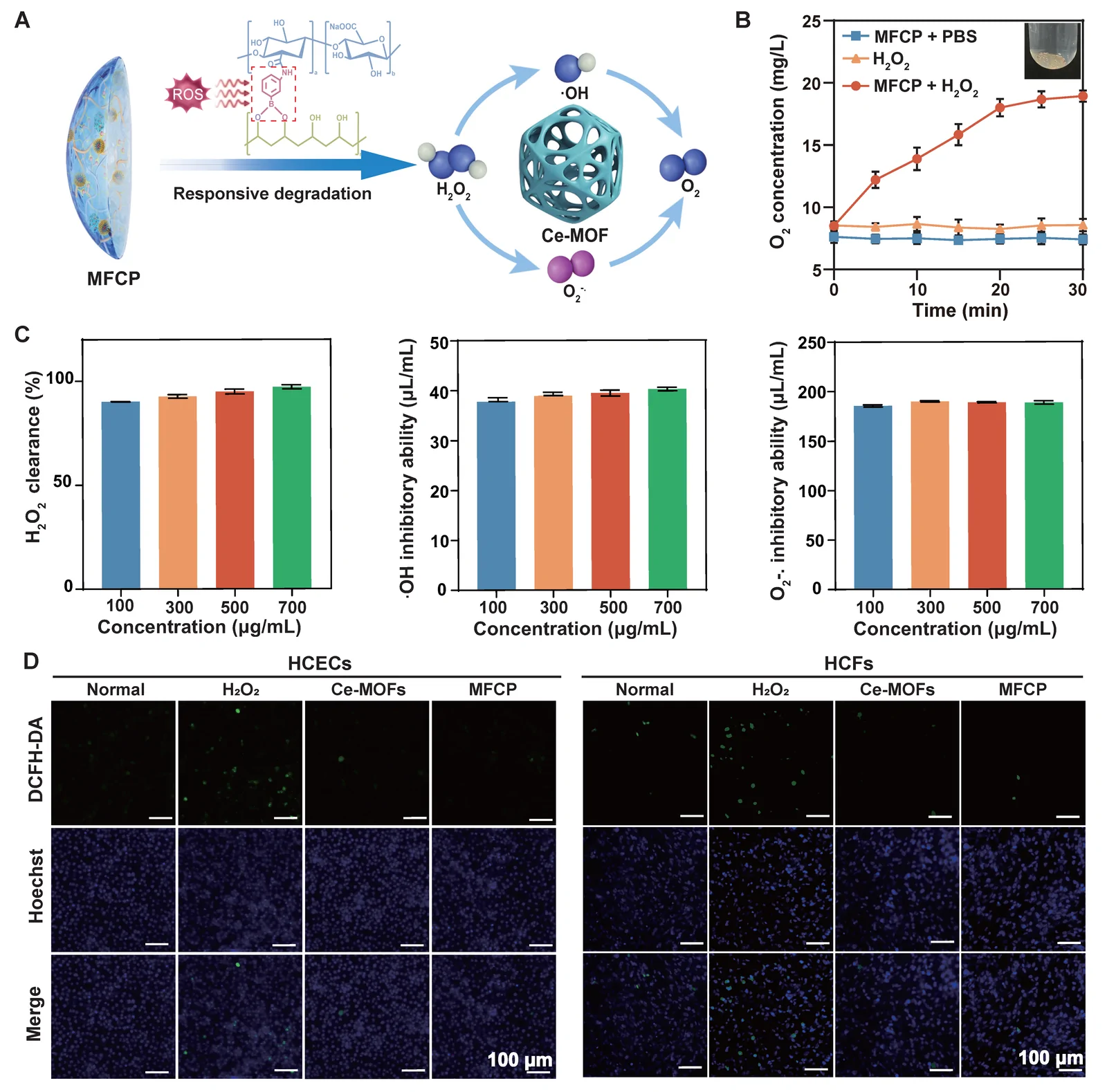
ROS-scavenging activity of MFCP. (A) Schematic of ROS-responsive MFCP degradation and ROS elimination by Ce-MOF. (B) Oxygen generation from MFCP upon H_2_O_2_ exposure. (C) ROS scavenging activities of Ce-MOFs against H_2_O_2_, ·OH, and O_2_⁻˙. (D) Intracellular ROS in cells exposed to Ce-MOFs or MFCP.

The antioxidant mechanism of MFCP involves ROS-responsive cleavage of borate ester bonds and subsequent release of Ce-MOFs, which catalyze the conversion of H_2_O_2_, O_2_⁻˙, and ·OH into molecular oxygen (Figure 5A). Incubation of MFCP with H_2_O_2_ generated oxygen in a time-dependent manner, confirming its catalytic decomposition of H_2_O_2_ (Figure 5B). The ROS-elimination activity of Ce-MOFs was quantified using chemical assay kits (Figure 5C). At 500 μg/mL, Ce-MOFs showed 95.68 ± 1.26% H_2_O_2_ elimination in a peroxidase-mimetic assay, along with superoxide dismutase-like (189.12 ± 0.64 μL/mL) and hydroxyl radical scavenging (39.72 ± 0.63 μL/mL) activities. A cellular oxidative stress model was established by exposing HCECs and HCFs to 100 μM H_2_O_2_. Fluorescence microscopy showed strong green fluorescence in H_2_O_2_-treated cells, indicating elevated ROS levels (Figure 5D). Pretreatment with Ce-MOFs or MFCP extract reduced fluorescence to near-baseline levels (Figure S9). These results confirm the ROS-scavenging ability of Ce-MOFs, thus mitigating oxidative stress.

### *In vitro* antibacterial activity

**Figure 6:**
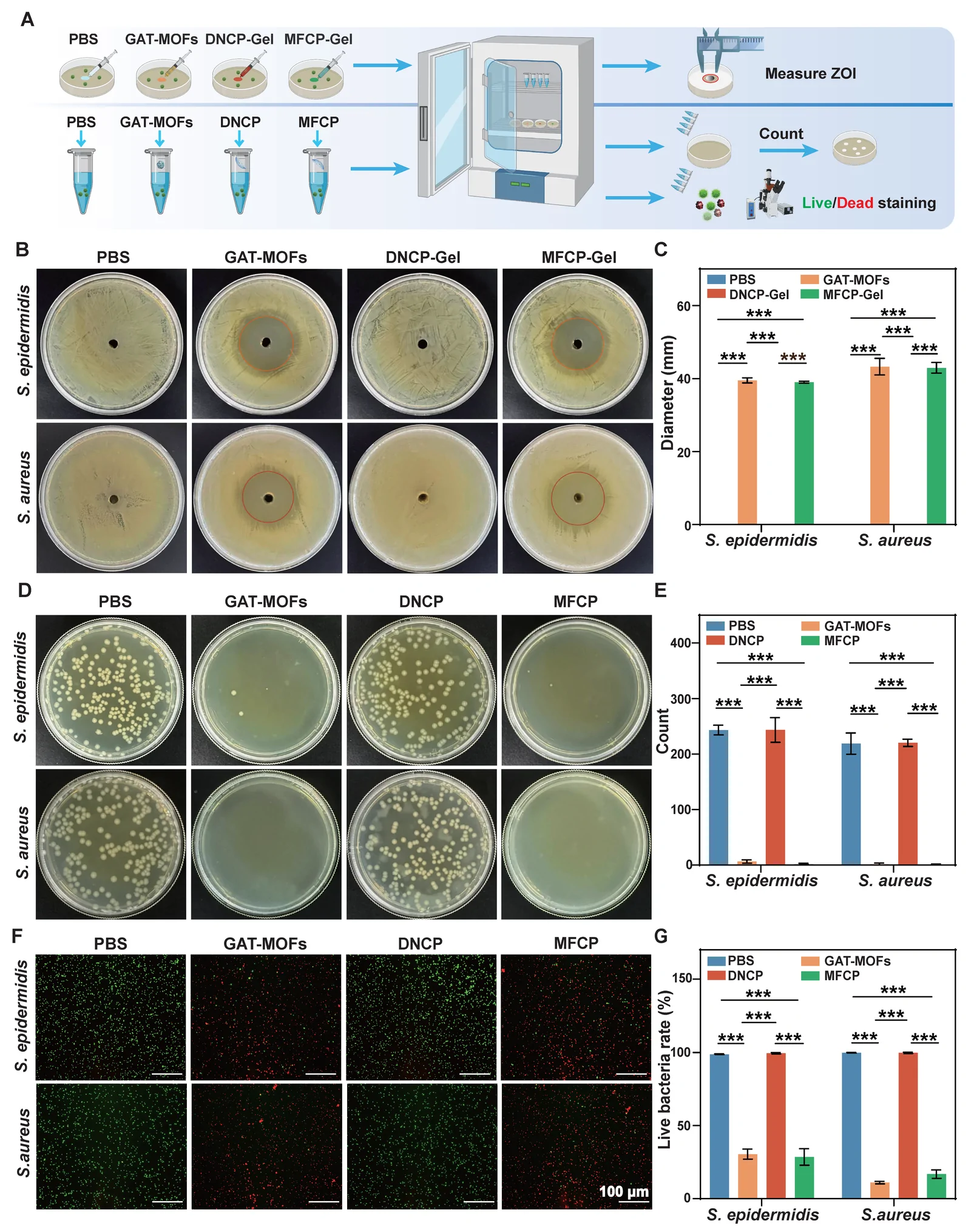
*In vitro* antibacterial activity of MFCP. (A) Schematic of the antibacterial evaluation methods. (B) ZOI images and (C) inhibition zone diameters. (D) Colony images and (E) CFU counts. (F) Live/dead staining, and (G) fluorescence intensity quantification. All groups were respectively treated with PBS, GAT-MOFs, DNCP, or MFCP. *P < 0.05, **P < 0.01, ***P < 0.001.

We evaluated the antibacterial activity of MFCP using zone of inhibition (ZOI), colony counting, and live/dead staining assays (Figure 6A) 18, 50, 51. GAT-MOFs and MFCP produced distinct inhibition zones against* S. epidermidis* and *S. aureus*, but the PBS and DNCP groups showed none (Figure 6B). Against *S. epidermidis*, the ZOIs were 39.53 ± 0.77 mm for GAT-MOFs and 39.01 ± 0.31 mm for MFCP. Against *S. aureus*, the ZOIs were 43.27 ± 2.51 mm for GAT-MOFs and 42.96 ± 1.69 mm for MFCP (Figure 6C). All values exceeded those of the controls. Free GAT solution and GAT-MOFs were compared at equivalent GAT concentrations (Figure S10). No significant difference was found, indicating that GAT loading preserved its antibacterial activity.

Colony counting assays showed reduced CFU numbers and sizes after GAT-MOF or MFCP treatment (Figure 6D). The PBS and DNCP groups exhibited dense bacterial growth, confirming the bactericidal activity of the drug-loaded formulations (Figure 6E). Live/dead staining further visualized bacterial viability. The PBS and DNCP groups showed predominantly green fluorescence, indicating high survival. The GAT-MOFs and MFCP groups exhibited strong red fluorescence, corresponding to extensive bacterial death (Figure 6F). Quantitative fluorescence analysis confirmed higher antibacterial rates for both materials against both strains (Figure 6G).

### *In vitro* antifungal activity

**Figure 7:**
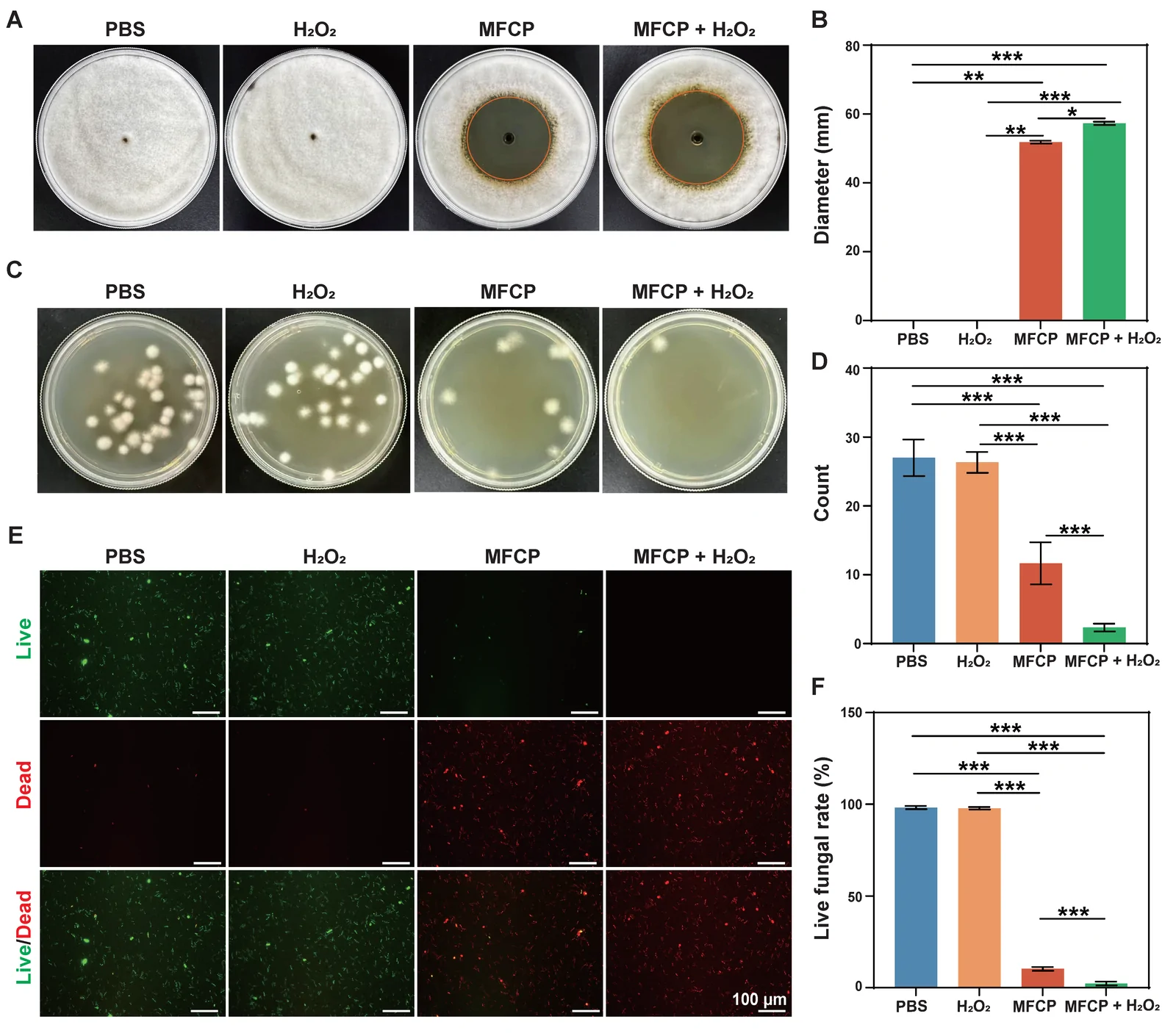
*In vitro* antifungal activity of MFCP. (A) ZOI images and (B) inhibition zone diameters. (C) Colony images and (D) CFU counts. (E) Live/dead staining and (F) fluorescence intensity quantification. All groups were treated with PBS, H_2_O_2_, MFCP, or MFCP + H_2_O_2_. *P < 0.05, **P < 0.01, ***P < 0.001.

We evaluated the antifungal activity of MFCP in the absence and presence of H_2_O_2_ (Figure 7A). H_2_O_2_ alone showed no antifungal activity. MFCP alone and MFCP + H_2_O_2_ (100 µM) produced inhibition zones of 51.83 ± 0.47 mm and 57.30 ± 0.50 mm, respectively (Figure 7B). Colony counting assays showed reduced fungal growth with MFCP alone, and nearly abolished colony formation with MFCP + H_2_O_2_ (Figures 7C-D). Live/dead staining showed a shift from green (live) to red (dead) fluorescence after MFCP treatment, with further increase upon H_2_O_2_ cotreatment (Figures 7E-F). The enhanced antifungal activity of MFCP + H_2_O_2_ likely arises from exogenous H_2_O_2_ accelerating MFCP degradation, leading to increased VCZ release and stronger antifungal efficacy.

### *In vivo* therapeutic efficacy of MFCP against fungal keratitis

**Figure 8:**
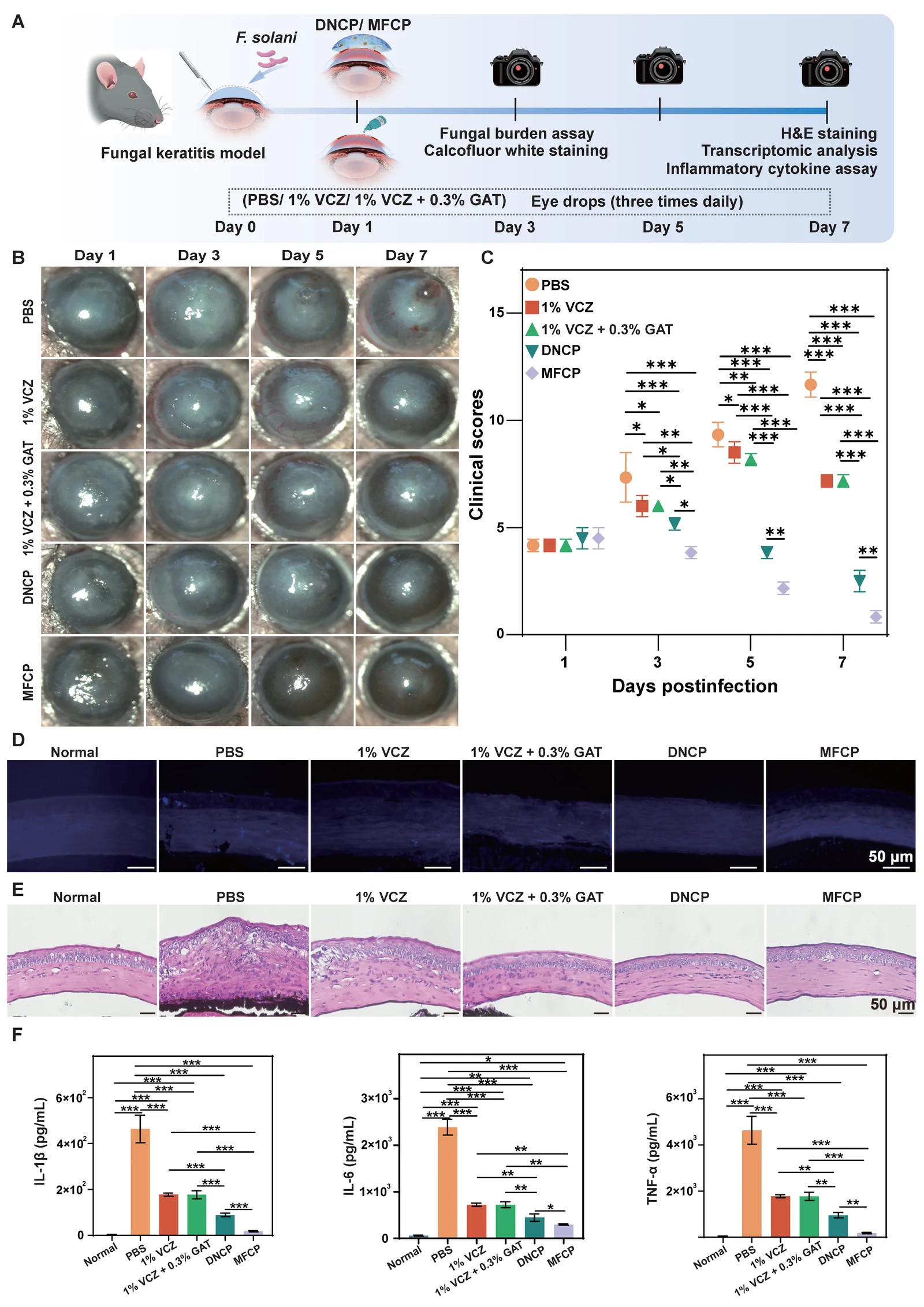
*In vivo* therapeutic efficacy of MFCP against fungal keratitis. (A) Treatment timeline. (B) Representative corneal images and (C) clinical scores during the follow-up period after infection. (n = 6). (D) Calcofluor white staining of corneas on day 3 (blue indicates fungal hyphae) (n = 3). (E) H&E staining of corneas and (F) corneal inflammatory cytokine levels on day 7 (n = 3). *P < 0.05, **P < 0.01, ***P < 0.001.

*In vivo* antifungal efficacy of MFCP was evaluated in a mouse model of fungal keratitis (Figure 8A). We monitored corneal changes in different treatment groups by slit-lamp imaging on days 1, 3, 5, and 7 after infection (Figure 8B). MFCP was the only treatment that kept corneas near-normal throughout the 7-day period, with only mild opacity at day 3 and minimal residual changes by day 7. In the PBS group, marked opacity and edema appeared by day 3 and worsened to hypopyon, neovascularization, and ulceration by day 7. 1% VCZ and 1% VCZ + 0.3% GAT exhibited modest antifungal effects, with little difference between them. DNCP reduced edema and neovascularization more effectively than the drug treatments, though it still didn't match MFCP. MFCP maintained the lowest clinical severity scores throughout the 7-day treatment period (Figure 8C).

We assessed corneal fungal burden by CFU counting after MFCP treatment. Compared with day 1, fungal colonies were almost undetectable by day 3 (Figure S11). Calcofluor white staining on day 3 revealed that hyphae were rarely detected in the MFCP and DNCP groups (Figure 8D). VCZ and VCZ + GAT groups showed some reduction compared with the PBS group, but hyphae were still present. MFCP and DNCP showed comparable efficacy in reducing hyphal invasion, with depths of 21.2 ± 3.1 μm and 23.4 ± 4.2 μm, respectively (Figure S12). By contrast, hyphal invasion was deeper in the other groups: PBS (146.4 ± 3.7 μm), VCZ (118.6 ± 1.7 μm), and VCZ + GAT (123.1 ± 1.4 μm) (Figure S12).

We evaluated the impact of different treatments on corneal structure and inflammation using H&E staining (Figure 8E). MFCP-treated corneas retained complete tissue structure and barely any infiltrating immune cells. VCZ and VCZ + GAT groups had less edema and fewer infiltrates than the PBS group, but considerable pathology persisted. These changes were milder in the DNCP group, though MFCP group remained the closest to normal corneal morphology. These findings indicate that MFCP effectively alleviated inflammation, supported by molecular analysis of proinflammatory cytokines in corneal tissues (Figure 8F).

### Transcriptomic profiling of MFCP-treated corneas

**Figure 9:**
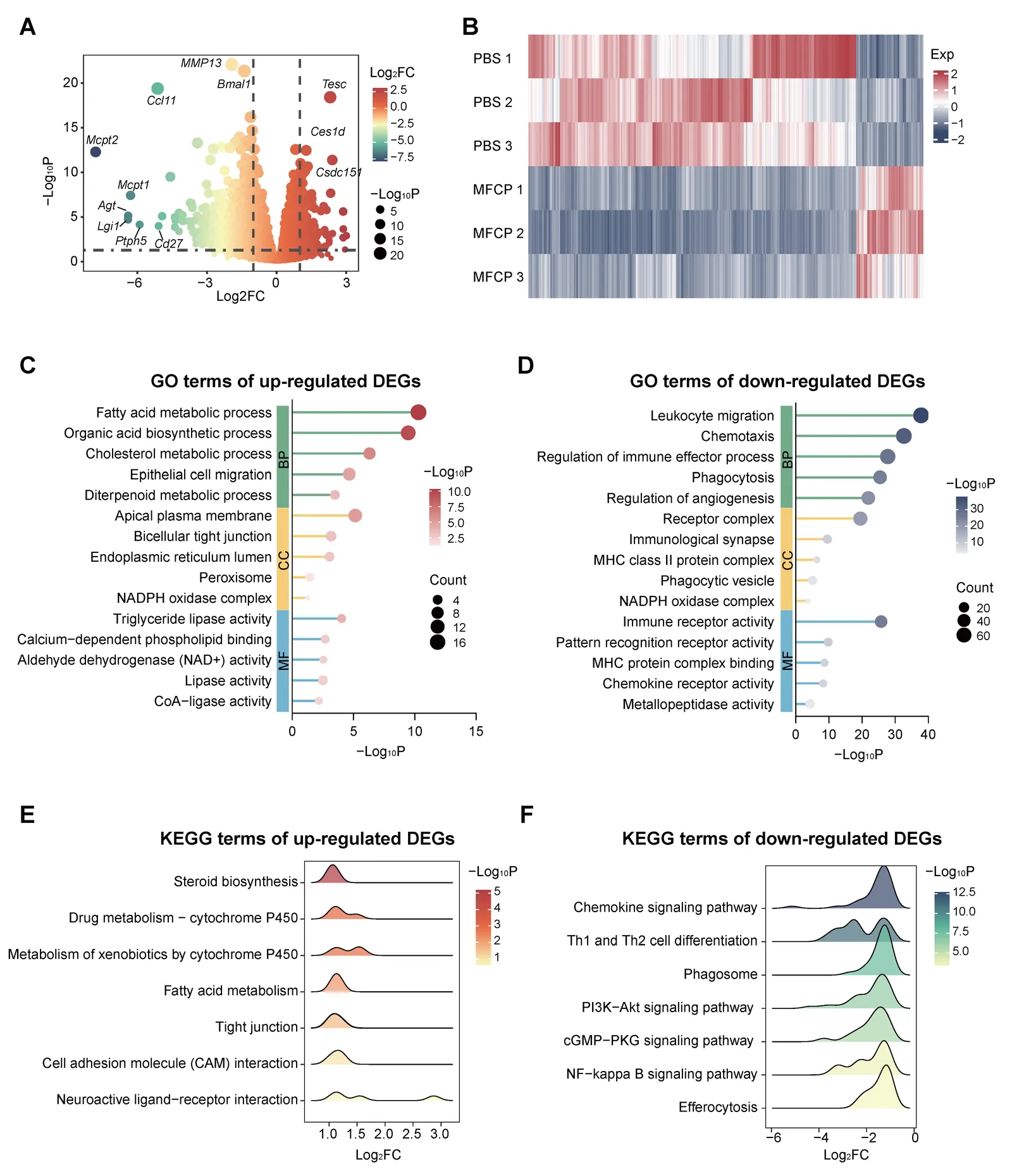
Transcriptomic profiling of MFCP-treated corneas (n = 3). (A) Volcano plot of DEGs between PBS and MFCP groups. (B) Heatmap of DEGs. (C, D) GO enrichment analysis of the upregulated and downregulated DEGs. (E, F) KEGG pathway enrichment analysis of the upregulated and downregulated DEGs.

We performed RNA sequencing on corneal tissues harvested on day 7 (Figures 9A-B). The MFCP group yielded 917 DEGs relative to the PBS group. Among these, 156 genes were upregulated and 761 downregulated. Genes involved in extracellular matrix remodeling (e.g., *Mmp13*) and biological rhythms (e.g., *Bmal1*) were upregulated. Inflammatory chemotaxis genes such as *Ccl11* were downregulated 52-54.

GO enrichment analysis of DEGs showed that upregulated genes were associated with metabolic and structural repair pathways, including fatty acid metabolism, cholesterol metabolism, epithelial cell migration, and tight junction (Figure 9C). Downregulated genes were linked to immune and inflammatory pathways, such as leukocyte migration, chemotaxis, immune effector regulation, and phagocytosis (Figure 9D). KEGG pathway analysis gave similar results (Figures 9E-F). Upregulated pathways included steroid biosynthesis, fatty acid metabolism, and tight junction. Downregulated pathways included chemokine, NF-κB, and PI3K-Akt signaling 54, 55.

MFCP treatment inhibited key proinflammatory pathways and genes, likely reflecting its dual anti-inflammatory design. Ce-MOFs scavenge ROS and limit NF-κB activation; voriconazole controls infection and eliminates persistent immune stimulation. These actions may promote inflammation resolution. MFCP also upregulated genes involved in lipid metabolism, cell migration, and cell junctions, suggesting a role in tissue repair. Sodium alginate from MFCP degradation may provide repair signals and promote epithelial regeneration and barrier restoration.

## Conclusions

In this study, we developed a feedback-regulated hydrogel corneal micropatch via 3D printing for the integrated therapy of fungal keratitis. The micropatch was printed using a bioink containing dynamic boronate ester bonds, enabling closed-loop feedback regulation of on-demand drug release and hydrogel degradation in response to ROS levels in the infected microenvironment. By integrating antifungal, antibacterial, antioxidant, and mechanical support functions, the MFCP demonstrated superior therapeutic efficacy compared to conventional eye drops, offering a novel feedback-regulated and multifunctional platform for managing fungal keratitis.

## Supplementary Material

Supplementary materials and figures.

## Data Availability

All data needed to evaluate the conclusions in the paper are present in the paper and the Supplementary Materials. Additional data related to this paper may be requested from the authors.
