# Supplementary material for: Feedback-regulated 3D-printed hydrogel micropatch for integrated therapy of fungal keratitis
Source: Theranostics. 2026 Aug 24;16(15):8827–43. doi: 10.7150/thno.134232 (PMC13549379; doi:10.7150/thno.134232)

Supplementary material

Feedback-regulated 3D-printed hydrogel micropatch for integrated therapy of fungal keratitis

Yujie Liu [1, 2, *], Li Ma [2, *], Guodong Fang [3], Tan Li [1], Hongran Zhao [1], Ting Wu [1, 2], Jiajia Zhang [1, 2], Xia Qi [1], Xiangyue Hu [1], Hongwei Wang [1], Chao Yang [3, ✉], Depeng Shi [1, ✉]

[1] Eye Institute of Shandong First Medical University, State Key Laboratory Cultivation Base, Shandong Provincial Key Laboratory of Ophthalmology, Qingdao Eye Hospital of Shandong First Medical University, Qingdao 266071, China.

[2] Department of Ophthalmology, First Affiliated Hospital of Dalian Medical University, Dalian 116011, China.

[3] College of Materials Science and Engineering, Qingdao University, Qingdao, Shandong, 266071, China.

* These authors contributed equally to this work.

✉ Corresponding authors: Depeng Shi, e-mail: dpshi@email.sdfmu.edu.cn; Chao Yang, e-mail: yangchao@qdu.edu.cn.

Figure S1. Scanning electron microscopy (SEM) image and corresponding energy-dispersive spectroscopy (EDS) elemental mapping images of C, N, O, and Ce of cerium-based metal-organic frameworks (Ce-MOFs).

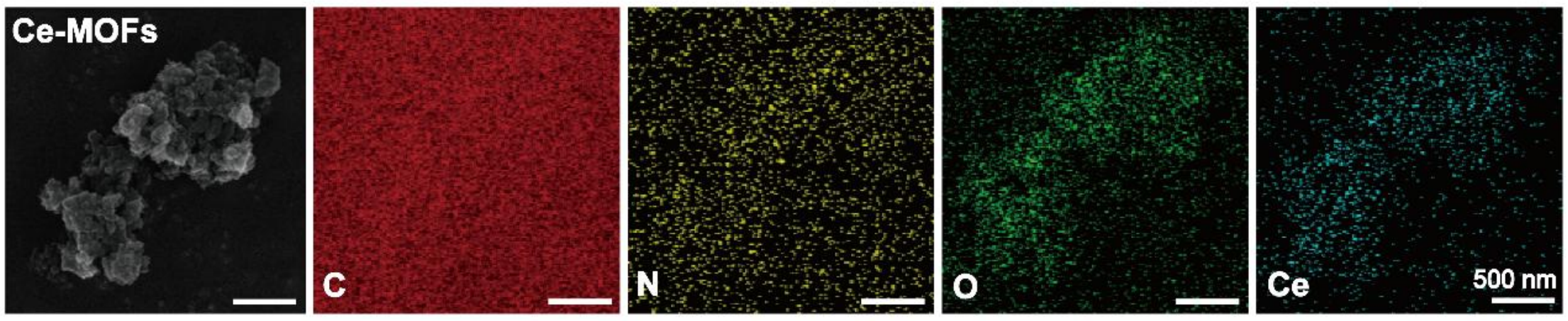

Figure S2. Zeta potential distribution map of Ce-MOFs.

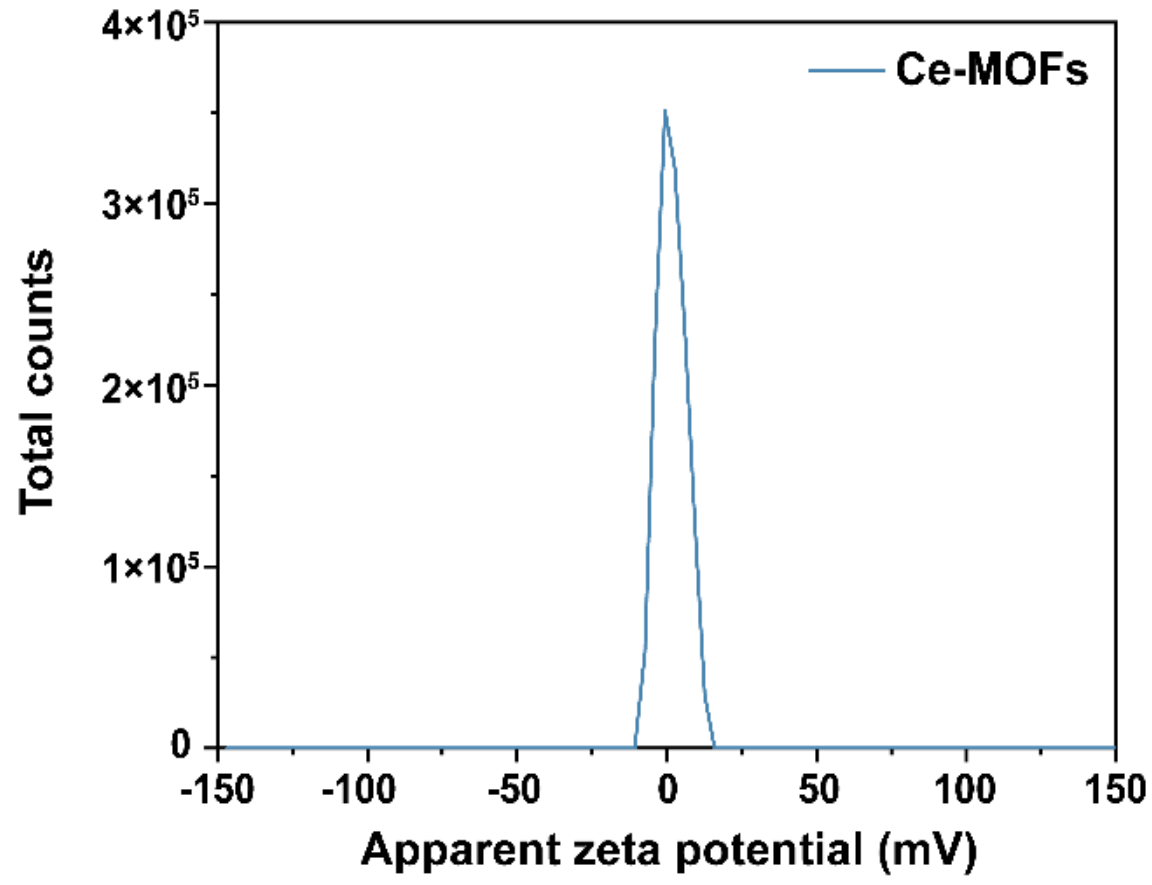

Figure S3. The storage moduli (G′) and loss moduli (G″) of F127DA-Gel, DNCP and MFCP.

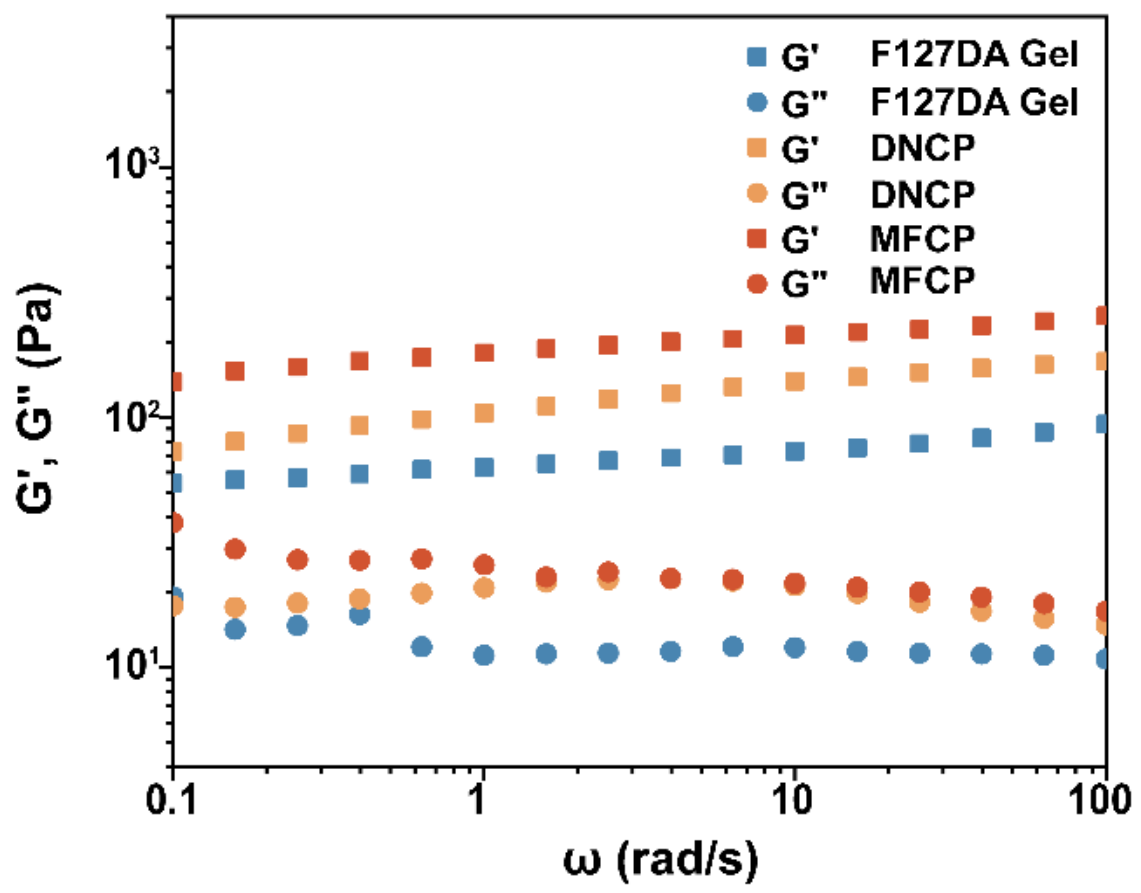

Figure S4. Application of MFCP based on corneal curvature via 3D printing in different animal corneas. (A) Application on the C57BL/6J mouse cornea. (B) Application on the New Zealand white rabbit cornea.

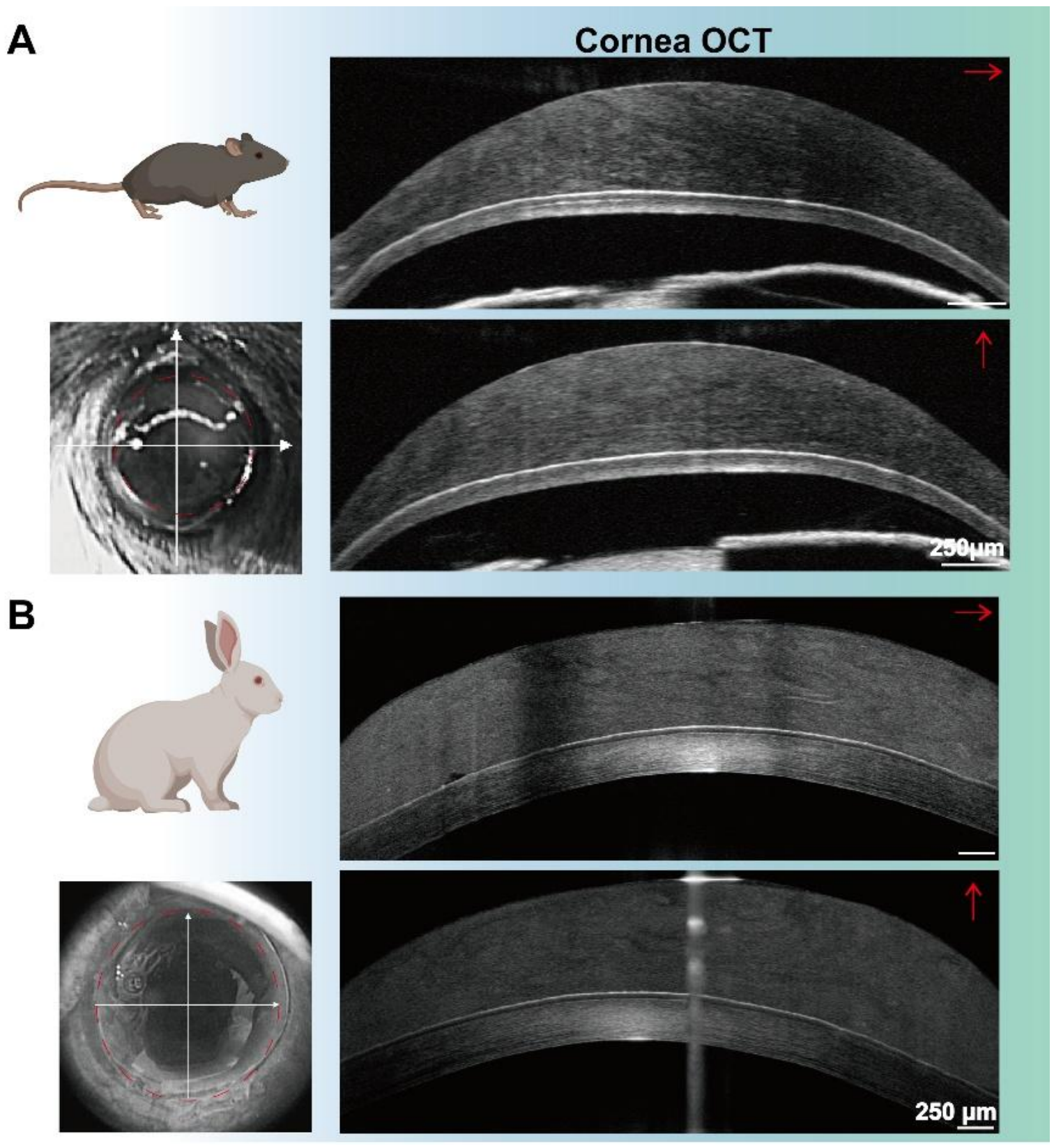

Figure S5. The moisture content of F127DA Gel, DNCP and MFCP.

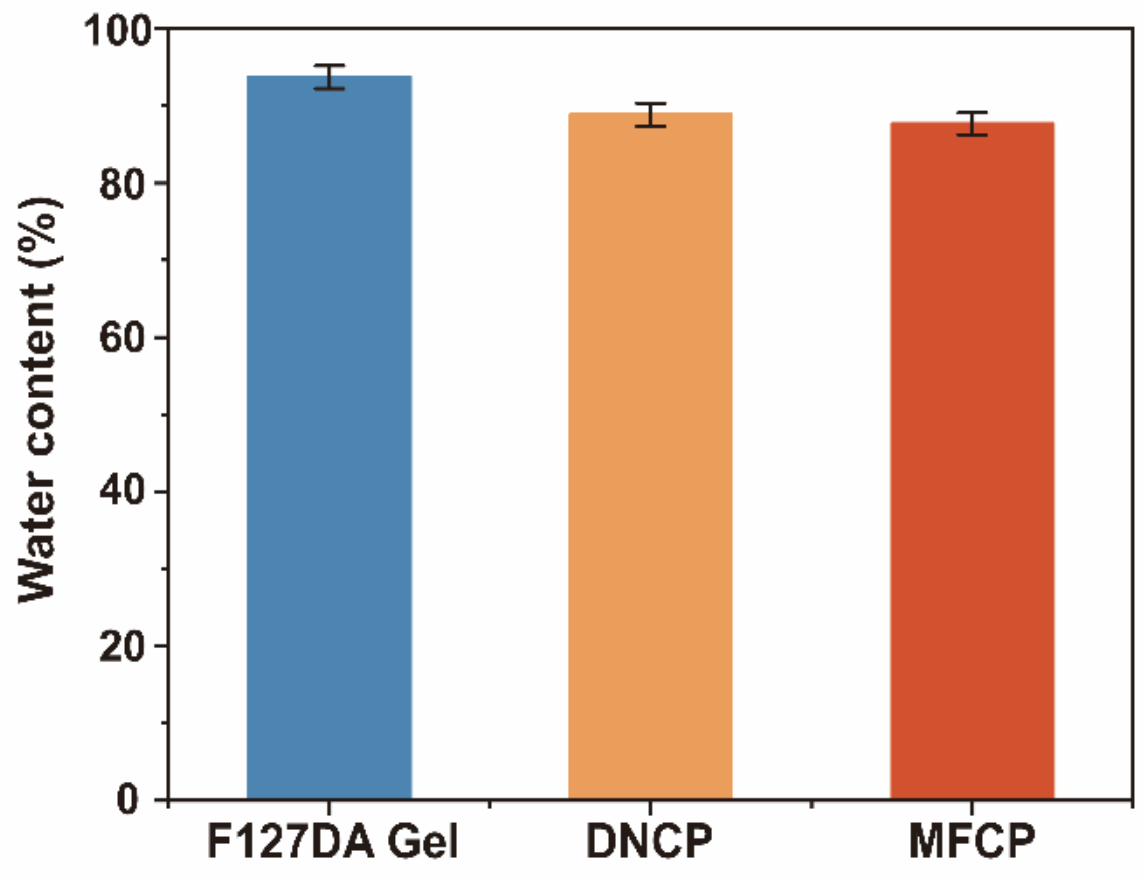

Figure S6. Quantitative analysis of the live/dead staining fluorescence on HCECs and HCFs was performed using ImageJ software.

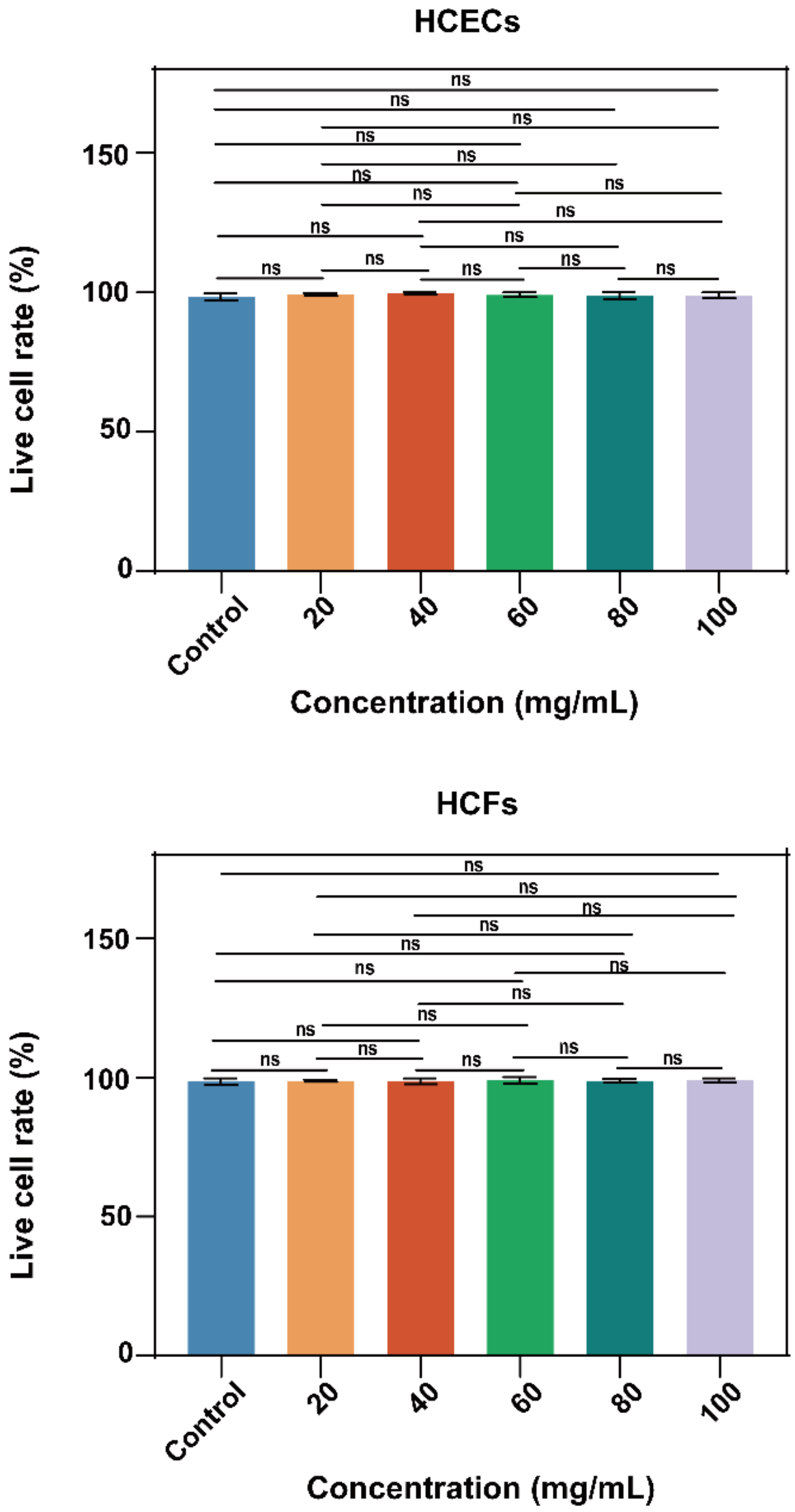

Figure S7. Quantitative real-time PCR was used to quantitatively analyze the mRNA expression levels of (A) α-SMA and (B) TGF-β_1.

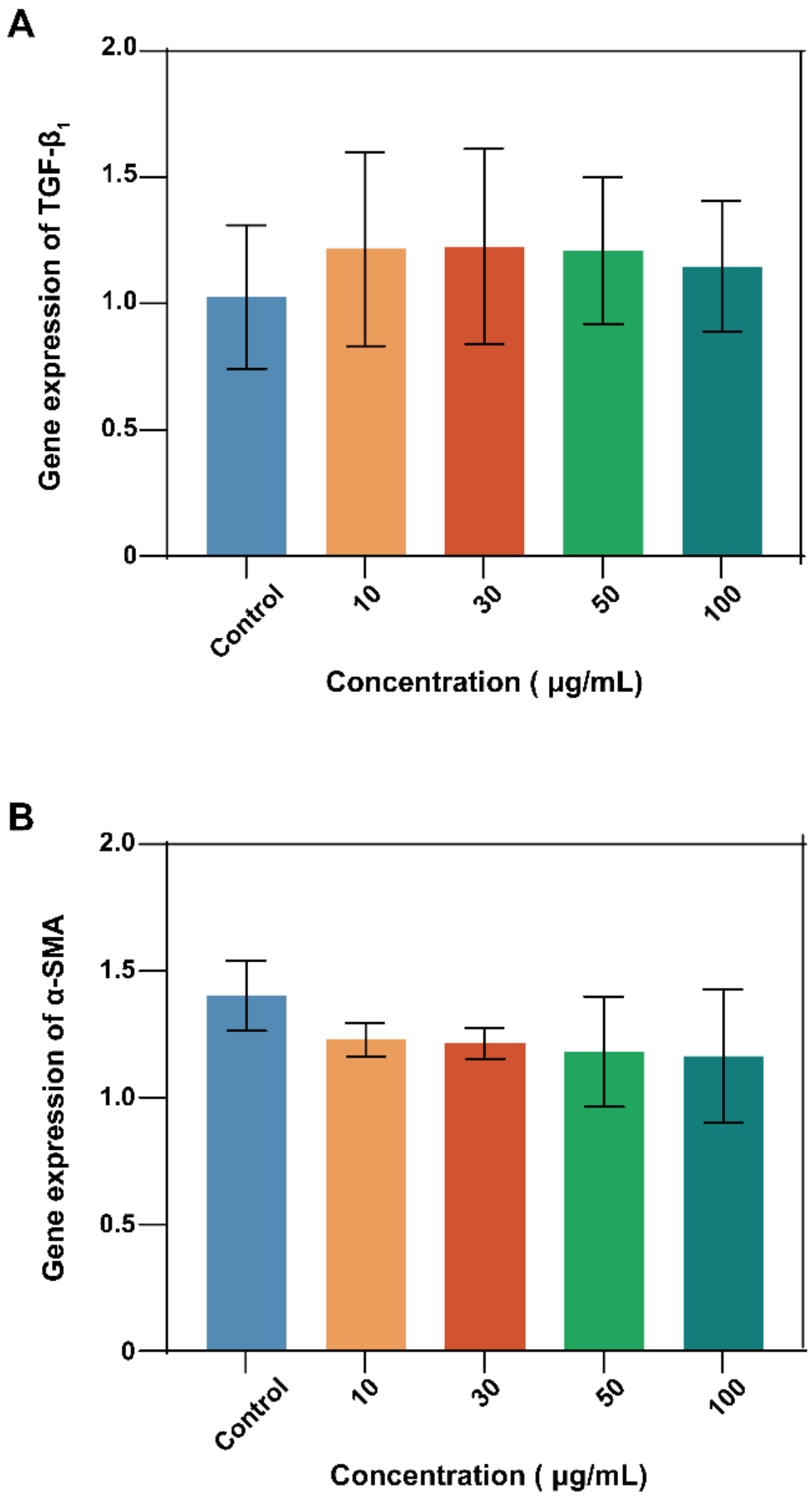

Figure S8. The bright-field image of the mouse cornea at 0, 24, and 48 h after treatment with PBS, 1% VCZ, GAT-MOFs, DNCP, or MFCP (n = 3).

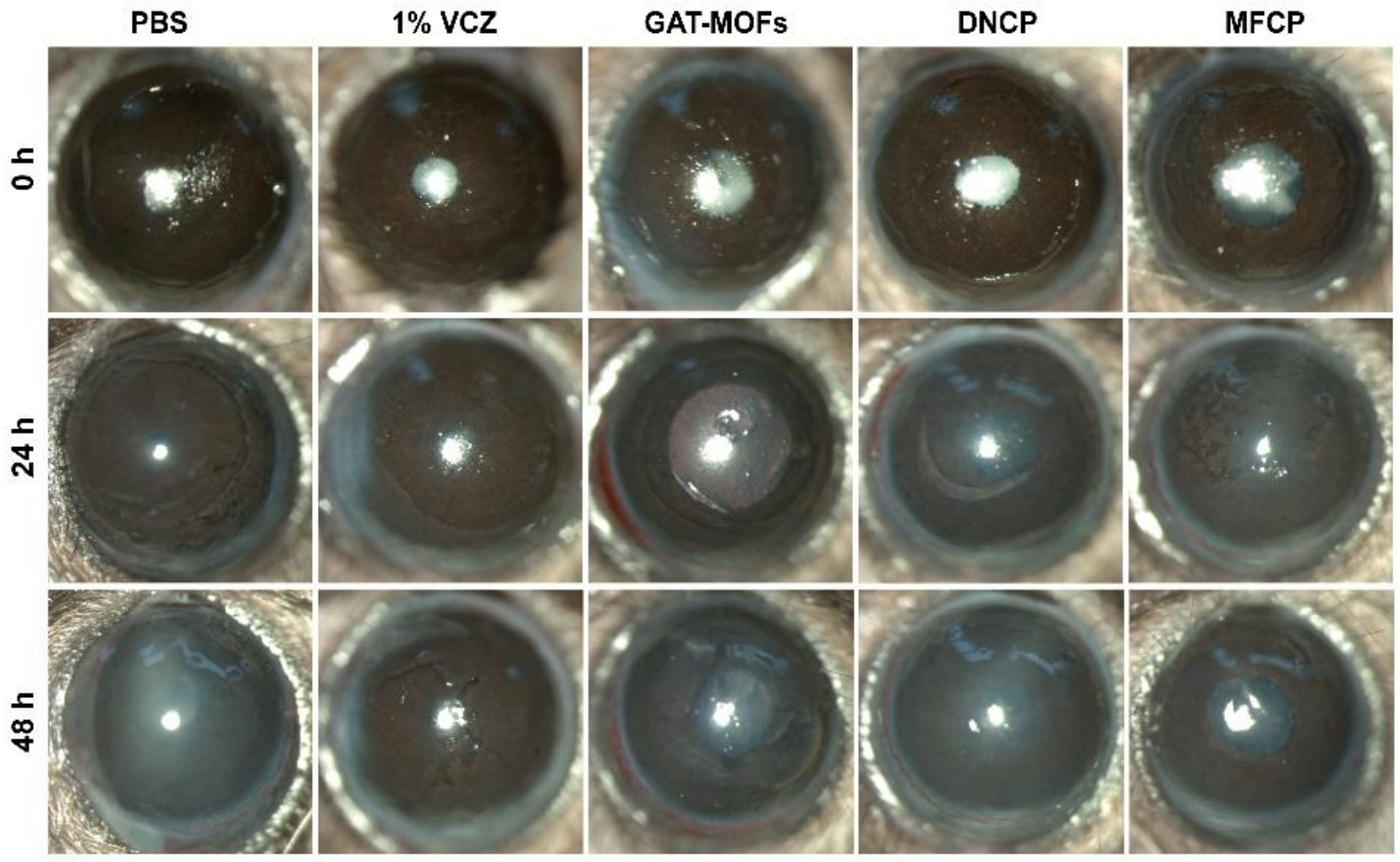

Figure S9. Quantitative analysis of ROS clearance fluorescence in HCECs and HCFs was conducted using ImageJ software.

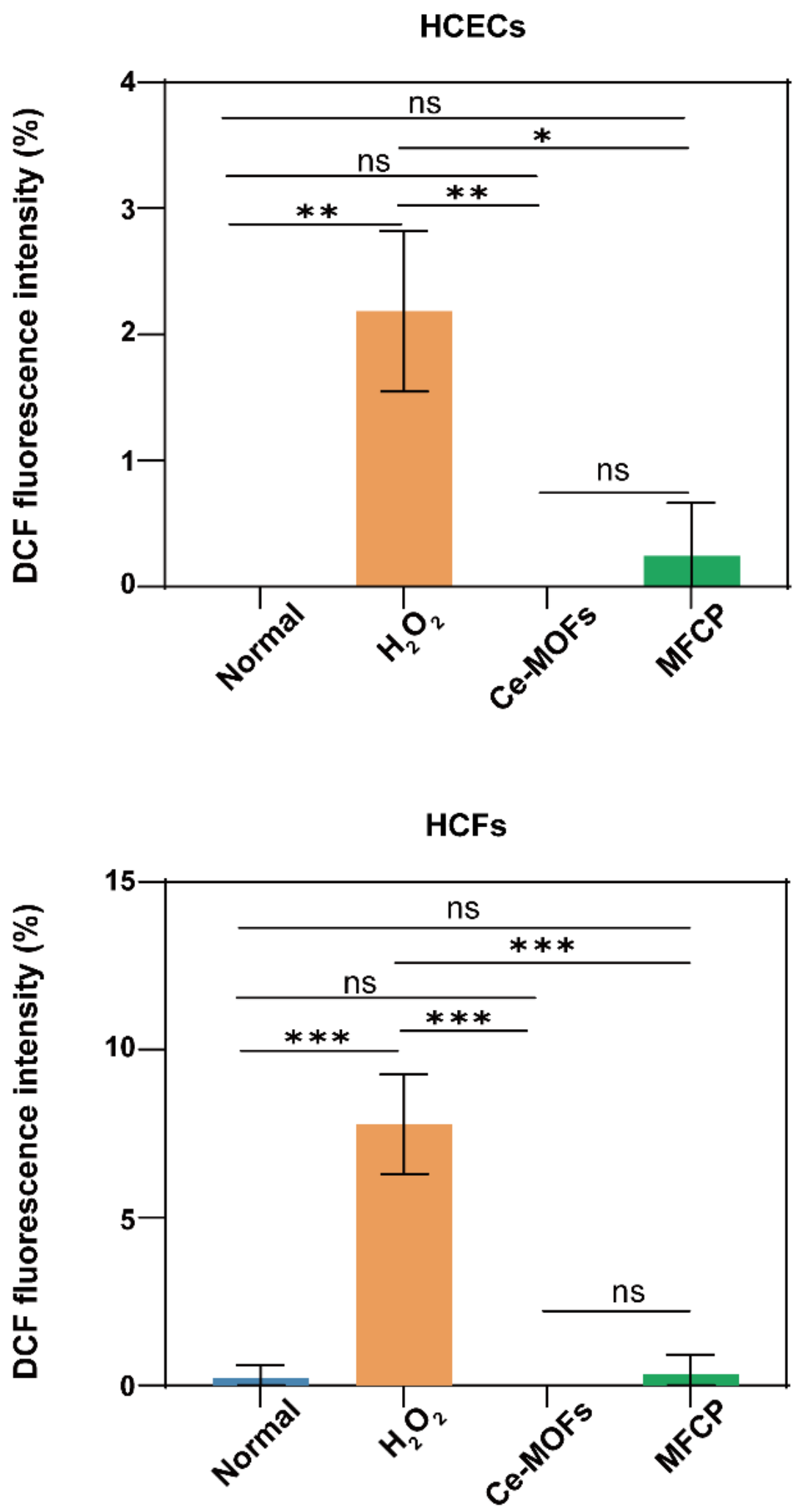

Figure S10. (A) Inhibition zone diameters against *S. epidermidis* and *S. aureus* for free GAT solution, (B) quantitative analysis of inhibition zone diameters between GAT and GAT-MOFs.

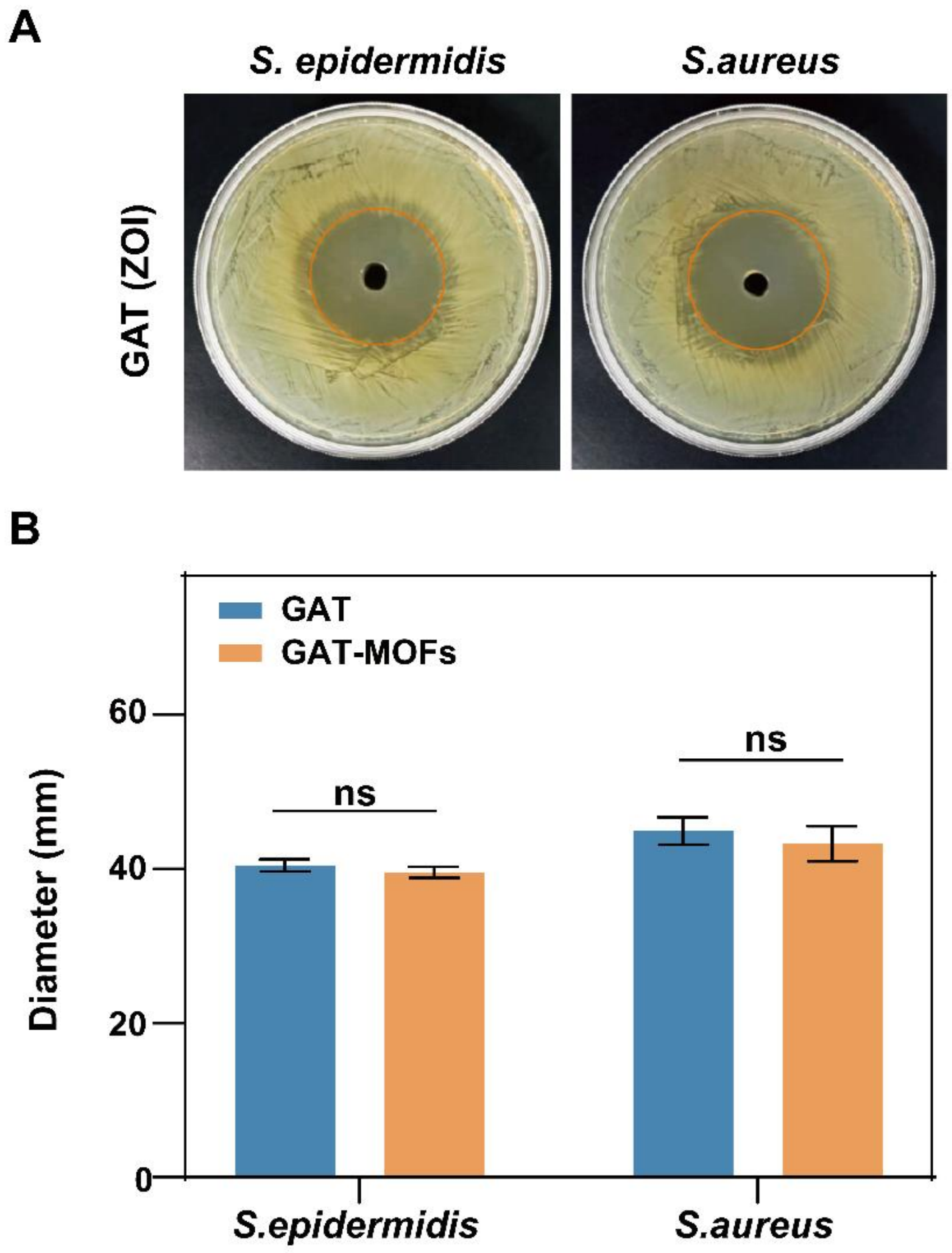

Figure S11. Fungal burden kinetics in MFCP-treated mouse corneas. Corneas were collected on days 1, 3, 5, and 7 post-infection (n = 3). (A) Images showing fungal colonies from the colony counting assay, accompanied by (B) statistical analysis of CFUs.

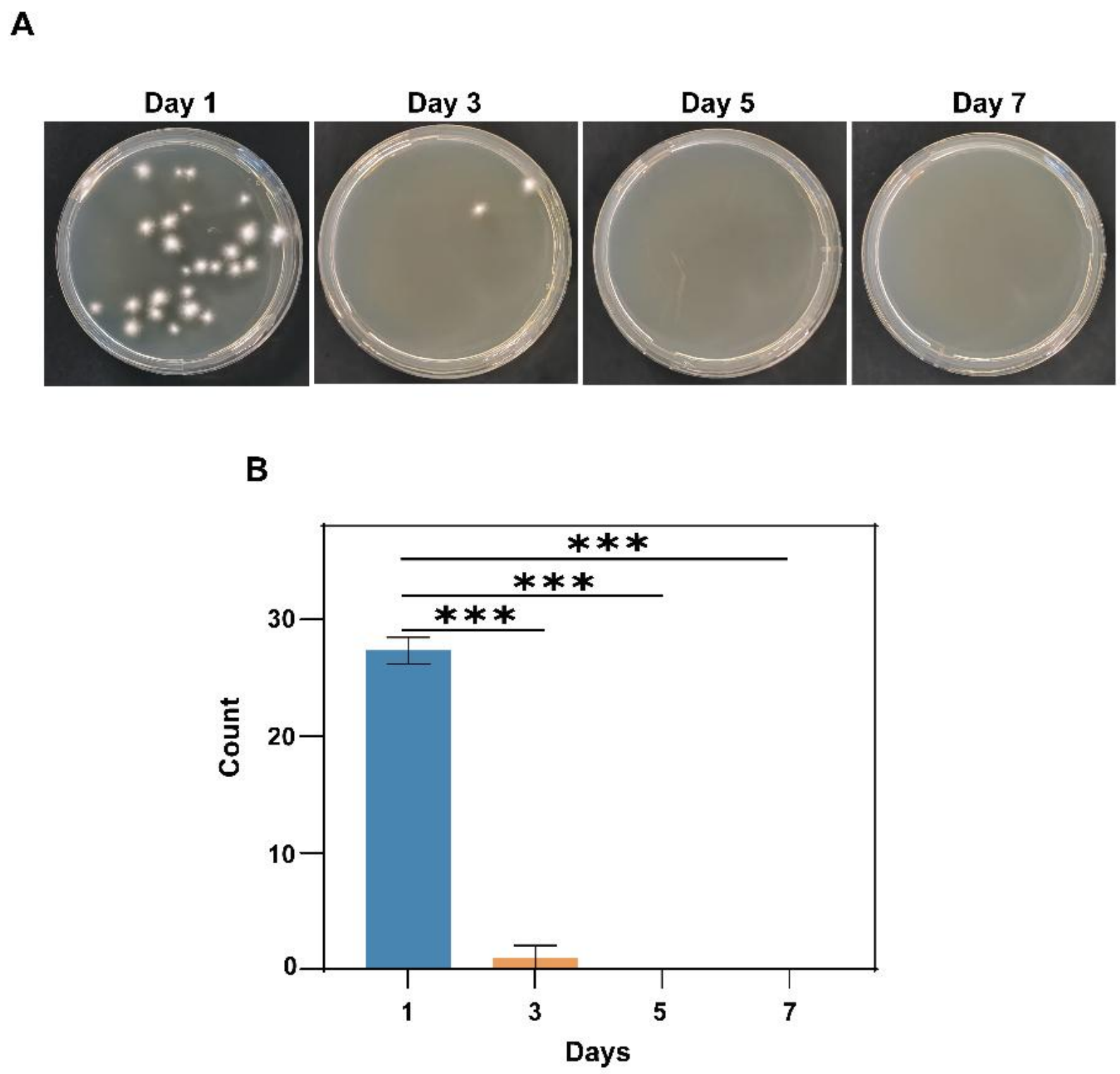

Figure S12. Fungal hyphal penetration depth in corneal stroma on day 3 post-infection.

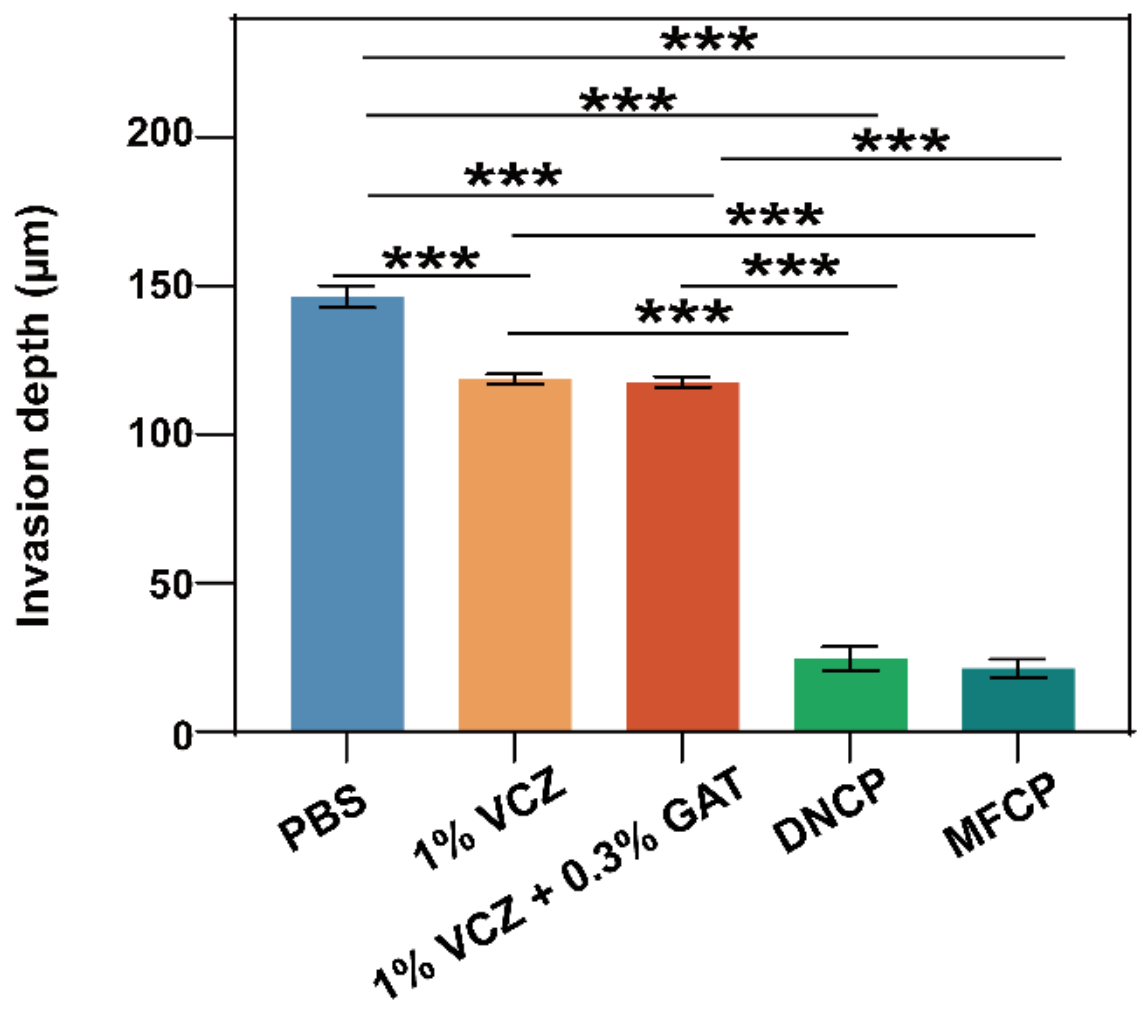

Supplement: Supplementary file 1 [file thnov16p8827s1.pdf]
